# Comparative genome analysis reveals niche-specific genome expansion in *Acinetobacter baumannii* strains

**DOI:** 10.1371/journal.pone.0218204

**Published:** 2019-06-13

**Authors:** Harshita Yakkala, Devyani Samantarrai, Michael Gribskov, Dayananda Siddavattam

**Affiliations:** 1 Dept. of Animal Biology, School of Life Sciences, University of Hyderabad, Hyderabad, India; 2 Department of Biological and Computer Sciences, Purdue University, West Lafayette, Indiana, United States of America; Tianjin University, CHINA

## Abstract

The nosocomial pathogen *Acinetobacter baumannii* acquired clinical significance due to the rapid development of its multi-drug resistant (MDR) phenotype. *A*. *baumannii* strains have the ability to colonize several ecological niches including soil, water, and animals, including humans. They also survive under extremely harsh environmental conditions thriving on rare and recalcitrant carbon compounds. However, the molecular basis behind such extreme adaptability of *A*. *baumannii* is unknown. We have therefore determined the complete genome sequence of *A*. *baumannii* DS002, which was isolated from agricultural soils, and compared it with 78 complete genome sequences of *A*. *baumannii* strains having complete information on the source of their isolation. Interestingly, the genome of *A*. *baumannii* DS002 showed high similarity to the genome of *A*. *baumannii* SDF isolated from the body louse. The environmental and clinical strains, which do not share a monophyletic origin, showed the existence of a strain-specific unique gene pool that supports niche-specific survival. The strains isolated from infected samples contained a genetic repertoire with a unique gene pool coding for iron acquisition machinery, particularly those required for the biosynthesis of acinetobactin. Interestingly, these strains also contained genes required for biofilm formation. However, such gene sets were either partially or completely missing in the environmental isolates, which instead harbored genes required for alternate carbon catabolism and a TonB-dependent transport system involved in the acquisition of iron via siderophores or xenosiderophores.

## Introduction

A surge in the number of multi-drug resistant (MDR) bacteria has increased the severity of many bacterial diseases. The number of infections caused by the MDR strains has almost quadrupled in the last two decades [1, 2]. Of these, the ESKAPE pathogens comprising of *Enterococcus faecium*, *Staphylococcus aureus*, *Klebsiella pneumoniae*, *Acinetobacter baumannii*, *Pseudomonas aeruginosa*, and *Enterobacter* species form the major proportion of MDR and extremely drug resistance (XDR) strains [3, 4]. The genus *Acinetobacter* are Gram-negative bacteria belonging to the class Gammaproteobacteria. Nearly 55 different species of *Acinetobacter* have been isolated from sources as varied as water [5], soil [6], hospitals [7, 8], body fluids [9, 10], and even body lice [11]. Almost all are known to cause human diseases. *Acinetobacter baumannii* is the predominant species of the genus and accounts for about 80% of reported *Acinetobacter* infections (https://www.cdc.gov/hai/organisms/acinetobacter.html), which range from pneumonia to serious blood or wound infections, soft tissue infections, and secondary meningitis [12–14]. The symptoms vary depending on the severity of the infection, but strains can also be asymptomatic, residing in tracheostomy sites or open wounds. *A*. *baumannii* strains are known for their genome plasticity and the ability to survive on abiotic surfaces [15, 16]. They acquire genes through conventional horizontal gene transfer (HGT), as well as through membrane vesicles [17–22]. Such robust gene acquisition contributes to the evolution of *A*. *baumannii* strains exhibiting MDR and XDR.

A total of 3539 *A*. *baumannii* genome sequences are available in the NCBI database. All these genomes display resistance traits acquired through HGT as part of the accessory genome. However, the genes that contribute to virulence are found as part of the core genome [12]. Genomes of *A*. *baumannii* strains have been compared to understand the molecular basis underlying their rapid acquisition of drug resistance genes. This comparative analysis of genomes revealed a surprisingly high degree of variations in the genomes of various strains, including single nucleotide polymorphisms (SNPs) as well as large DNA fragment variations [23]. Interestingly, in the AYE strain of *A*. *baumannii*, an 86 Kb resistance island (RI) was identified carrying 45 resistance genes. However, at the analogous position in the drug-sensitive SDF strain of *A*. *baumannii*, a 20 Kb genomic island (GI) devoid of RIs was detected, suggesting site-specific integration of resistance genes at this hotspot in the genome [24]. In addition to the RIs, diverse antibiotic resistance determinants were identified outside of the RIs. Antibiotic resistance gene-bearing integrons and *blaOXA*-23-containing transposon, Tn*2009* were detected in the genome of several *A*. *baumannii* isolates. Sequence similarity and phylogenetic analyses have revealed that the resistance genes found in *A*. *baumannii* have been acquired relatively recently from bacteria of the genera *Pseudomonas*, *Salmonella*, or *Escherichia* [25].

*A*. *baumannii* strains adapt to a variety of ecological niches. They survive in soil and water in a free-living state and can quickly adapt to the infectious mode as a lifestyle. Such robust survival strategies of *A*. *baumannii* strains suggest they have acquired the genetic repertoire allowing them to adapt quickly to changing environmental conditions. Unfortunately, few studies have been conducted to identify unique genes that contribute to niche-specific survival of *A*. *baumannii* strains [11]. The present study reports a complete genome sequence of *A*. *baumannii* DS002, which was isolated from pesticide-polluted agricultural soils. Comparison of its genome sequence with the complete genome sequences of *A*. *baumannii* strains available in the NCBI database revealed the existence of unique, niche-specific genes that contribute to the fitness and survival of the bacterium in a variety of different habitats.

## Materials and methods

### Genome sequencing, assembly, and annotation

*A*. *baumannii* DS002 was grown to early stationary phase in LB medium at 30°C and the genomic DNA was isolated using a QIAGEN Genomic-tip Kit. The quality and quantity of the isolated DNA were assessed using agarose gel electrophoresis and Qubit 3.0, which is a fluorometric instrument used for quantification of DNA, RNA, and protein. The isolated DNA was subjected to sequencing using a Pacific Biosciences (PacBio) single-molecule real-time (SMRT) cell RS II instrument (chemistry version P6-C4) at Nucleome Informatics, Hyderabad. A total of 89,614 reads were obtained, out of which, 86,803 reads were more than 500bps in length. The average read length was 5601bp. The genome assembly was done by the Canu 1.8 assembly pipeline using HISEA read overlapper [26, 27]. The Canu+HISEA process involved four basic steps of read error-correction, quality/adapter trimming, contig layout construction and consensus generation. Several iterations of the assembly pipeline were performed to determine the optimal parameters. About 80,184 reads survived the error correction and trimming steps, resulting in eight different assemblies. The assemblies were annotated using Prokka v1.12 [28] and Prodigal 2.6.1 [29] pipelines. Benchmarking Universal Single-Copy Orthologs (BUSCOs) from the gammaproteobacteria set was used as a standard to assess the quality of the assembly and annotation performed [30]. A circular map of the chromosome was generated using Arc with color tool (http://www.ige.tohoku.ac.jp/joho/gmProject/gmdownload.html). The bacterial culture was deposited in the Microbial Type Culture Collection center (MTCC), IMTECH, Chandigarh, India, and is available as *Acinetobacter* sp. DS002 MTCC11451.

### Bacterial pan-genome analysis (BPGA)

In order to identify strain-specific genomic features in a genome and determine the genomic diversity among the *A*. *baumannii* strains, the computational pipeline BPGA tool was used [31]. Although a total of 3539 *A*. *baumannii* genome sequences are available in the NCBI database most of them are incomplete and exist as scaffolds. In certain cases a complete genome sequence is available but there is an ambiguity with respect to the source of isolation. Therefore, such sequences were not considered for the pan-genome analysis. Only complete genome sequences of *A*. *baumannii* having information on the source of isolation were included in the analysis. This list comprises of 78 genome sequences, which includes the complete genome sequence of *A*. *baumannii* DS002 (Table 1). The full GenBank files of all 78 genomes downloaded from NCBI served as input for the BPGA analysis. BPGA further processed these files for orthologous cluster analysis and generated an input file containing a total of 281,137 annotated genes. This input file was subsequently used for clustering of genes into families by USEARCH with 50% sequence similarity as a cutoff. To avoid any bias during the sequential addition of genomes, 30 random permutations in the sequence/order of genomes added were carried out. The size of the pan-genome is represented by median values of the total number of distinct gene families and the core genome is represented by the number of shared gene families. Preliminary pan-genome profile analysis was performed to determine the frequency distribution of various gene families among the selected 78 complete genomes. Using the pan-genome sequence extraction module, core, accessory, and unique gene families were extracted. The exclusive gene family analysis module was used to identify homologous gene families that are distinctive for *A*. *baumannii* strains (i.e., unique genes or singletons). The pan-genome functional analysis module was used to find the Clusters of Orthologous Groups of proteins (COGs) and KEGG pathway distribution. Evolutionary analysis done by BPGA was based on concatenated core gene alignment using a binary pan-matrix file that depicts the presence or absence of the genes among the genomes. The pan-gene matrix was generated by calculating the contribution of the gene to the orthologous gene clusters. The core genome phylogenetic tree was constructed using protein sequences of 20 random orthologous gene clusters. MUSCLE (MUltiple Sequence Comparison by Log-Expectation) was used for initial multiple sequence alignment and the aligned sequences were then concatenated before constructing a neighbor-joining phylogenetic tree. To assess the genetic diversity among the genomes we performed an average nucleotide identity (ANI) analysis using the FastANI tool [32], which computes a pair-wise ANI value for the sample genome. These ANI values were then used to generate a heatmap using webserver heat mapper (http://www.heatmapper.ca) and to convert into a Euclidean distance matrix. This distance matrix was then uploaded to the web server DendroUPGMA (http://genomes.urv.cat/UPGMA/) [33] to generate a Newick format of the distance matrix. The Newick file is then used in MEGA7 [34] to view the UPGMA (unweighted pair group method with arithmetic mean) Tree based on ANI based Euclidian distance.

**Table 1:** List of 78 *A*. *baumannii* strains and source of isolation.

Genomic IDs	Strain	Source of isolation	Broader-Isolation Source
AP013357.1	NCGM-237	RTI/UTI/Blood	Blood
AP014649.1	IOMTU-433	Respiratory tracts	Tissue
CP000863.1	ACICU	Cerebrospinal fluid	Body fluids
CP024911.1	AB307_0294_2	Blood	Blood
CP001182.2	AB0057	Bloodstream	Blood
CP001921.1	1656–2	Hospital Setting- South Korea	Hospital
CP001937.1	MDR-ZJ06	Bloodstream	Blood
CP003500.1	MDR-TJ	Hospital setting	Hospital
CP003846.1	BJAB07104	Blood	Blood
CP003847.1	BJAB0715	Spinal fluid	Body fluids
CP003849.1	BJAB0868	Ascites	Body fluids
CP003856.1	TYTH-1	Bacteremia- Taiwan	Bacteremia
CP003967.2	D1279779	Bacteraemic infection of an indigenous Australian male	Bacteremia
CP006768.1	ZW85-1	Diarrheal patient feces	Body fluids
CP007535.2	AC29	Endotracheal Secretion	Body fluids
CP007577.1	AC30	Endotrachial secretion	Body fluids
CP007712.1	LAC-4	Outbreak	Outbreak
CP008706.1	AB5075-UW	Tibia/Osteomyelitis	Tissue
CP009256.1	AB031	Bloodstream	Blood
CP009257.1	AB030	Bloodstream	Blood
CP009534.1	AbH12O-A2	Outbreak in Spain	Outbreak
CP010397.1	6200	Body fluid	Body fluids
CP010779.1	XH386	Lower respiratory tract	Tissue
CP010781.1	A1	Nottingham University Hospital	Hospital
CP012006.1	Ab04-mff	Blood	Blood
CP012952.1	D36	Wound	Wound
CP013924.1	KBN10P02143	Pus	Body fluids
CP014215.1	YU-R612	Sputum	Sputum
CP014528.1	XH858	Sputum	Sputum
CP014539.1	XH859	Wound	Wound
CP014540.1	XH857	Sputum	Sputum
CP014541.1	XH856	Drainage fluid	Body fluids
CP015364.1	3207	Bronchial fluid	Body fluids
CP015483.1	ORAB01	Body fluid	Body fluids
CP016298.1	CMC-MDR-Ab59	Sputum	Sputum
CP017152.1	DU202	Clinical isolate	Hospital
CP017642.1	KAB01	Bronchial	Body fluids
CP017644.1	KAB02	Bronchial	Body fluids
CP017646.1	KAB03	Sputum	Sputum
CP017648.1	KAB04	Sputum	Sputum
CP017650.1	KAB05	Blood	Blood
CP017652.1	KAB06	Wound	Wound
CP017654.1	KAB07	Sputum	Sputum
CP017656.1	KAB08	Wound	Wound
CP018143.1	HRAB-85	Sputum	Sputum
CP018254.1	AF-401	Small colon	Tissue
CP018256.1	AF-673	Sputum	Sputum
CP018332.1	A1296	Sputum	Sputum
CP018421.1	XDR-BJ83	Human	Human
CP018664.1	ATCC-17978	Blood	Blood
CP020574.1	15A5	Pulmonary	Tissue
CP020578.1	SSA12	Blood	Blood
CP020579.1	SAA14	Blood	Blood
CP020581.1	SSMA17	Bronchial washing fluid	Body fluids
CP020584.1	JBA13	Sputum	Sputum
CP020586.1	CBA7	Sputum	Sputum
CP020590.1	15A34	Pulmonary	Tissue
CP020591.1	SSA6	Endotracheal aspirate	Body fluids
CP020592.1	USA2	Urine	Body fluids
CP020595.1	USA15	Sputum	Sputum
CP020597.1	HWBA8	Sputum	Sputum
CP020598.1	WKA02	Sputum	Sputum
CP021342.1	B8342	Blood	Blood
CP021347.1	B8300	Blood	Blood
CP021782.1	A85	Sputum	Sputum
CP024124.1	AYP-A2	Wound	Wound
CP024576.1	AbPK1	Sheep broncho-alveolar lavage	Tissue
CP024611.1	Ab4977	Sputum	Sputum
CP024612.1	Ab4653	Sputum	Sputum
CP024613.1	Ab4568	Sputum	Sputum
CP001172.1	AB307_0294	Blood	Blood
CP025266.1	SMC_Paed_Ab_BL01	Blood, Central line	Blood
CP027704.1	DS002	Soil	Soil
CU459141.1	AYE	Patient with Pneumonia & UTI	Human
CU468230.2	SDF	Body Louse	Environmental
LN865143.1	CIP70.10	Human skin	Human skin
LN868200.1	R2090	Tissue	Tissue
LN997846.1	R2091	Tissue	Tissue

### Comparative genomics

The primary objective of this study is to identify a niche-specific unique gene content(s) in *A*. *baumannii* strains. No environmental isolate other than DS002 exists among the 78 genome sequences included in the analysis. Therefore, we have considered the inclusion of the draft genome sequence of DSM30011 to gain insight into the unique gene content in the environmental isolate. The seventy-nine (including DSM30011) sequences of *A*. *baumannii* strains were considered for the comparative analysis of virulome, resistome, and genes involved in iron acquisition and metabolic pathways. We compiled a list of genes involved in virulence, resistance, iron acquisition, and carbon metabolism in *A*. *baumannii* from different literature studies as well as from the KEGG pathway tools (S1 File). These gene sets were then used as input to perform a Large-Scale BLAST Score Ratio (LS-BSR) analysis [35]. Principal Component Analysis (PCA) plot for LS-BSR output score matrix is generated by using the R package ggplot2 and scatterplot3d. Further, we set a BSR cut-off of 0.6 and above to determine the presence of genes and any value below 0.6 indicated their absence. The matrix file obtained from LS-BSR was used to generate heat maps and was further clustered hierarchically to provide a better understanding of the relationship between the strains using the R platform version 3.4.4 [36].

### Resistome and genomic island (GI) prediction

The Resistance Gene Identifier (RGI) software of CARD (The Comprehensive Antibiotic Resistance Database) was used for prediction of the resistome [37]. The presence of GI was predicted by IslandViewer 4 [38], which uses three prediction algorithms such as SIGI-HMM, IslandPath-DIMOB, and IslandPick to calculate codon usage, dinucleotide bias within a genome, and to generate a dataset of GIs and non-GIs from phylogenetically related organisms. The GIs predicted by at least one of the three algorithms is considered for detailed analysis. In order to gain better clarity on the existence of GI and their putative functions, only eleven genome sequences, derived from strains isolated from bacteremia (TYTH-1) (CP003856), hospital (MDR-TJ) (CP003500), body fluid (6200) (CP010397), sputum (HWBA8) (CP020597), wound (AYP-A2) (CP024124), tissue (IOMTU 433) (AP014649), blood (AB031) (CP009256), infectious outbreaks (LAC-4) (CP007712), humans (AYE) (CU459141.1), and environmental (SDF) (CU468230) samples were included along with DS002 genome sequence. These genome sequences were selected by taking one from each source of isolation, and which had the highest number of unique genes.

### Quantification of bacterial growth and biofilm formation

*A*. *baumannii* strains AYE and DS002 were grown either in LB medium or in minimal salt medium [39] supplemented with phenol (2 mM) as the sole source of carbon at 30°C. The biofilm formation of the strains AYE and DS002 was determined following standard procedures [40].

## Results

### Genomic size and GC content of *A*. *baumannii* DS002

**Fig 1:**
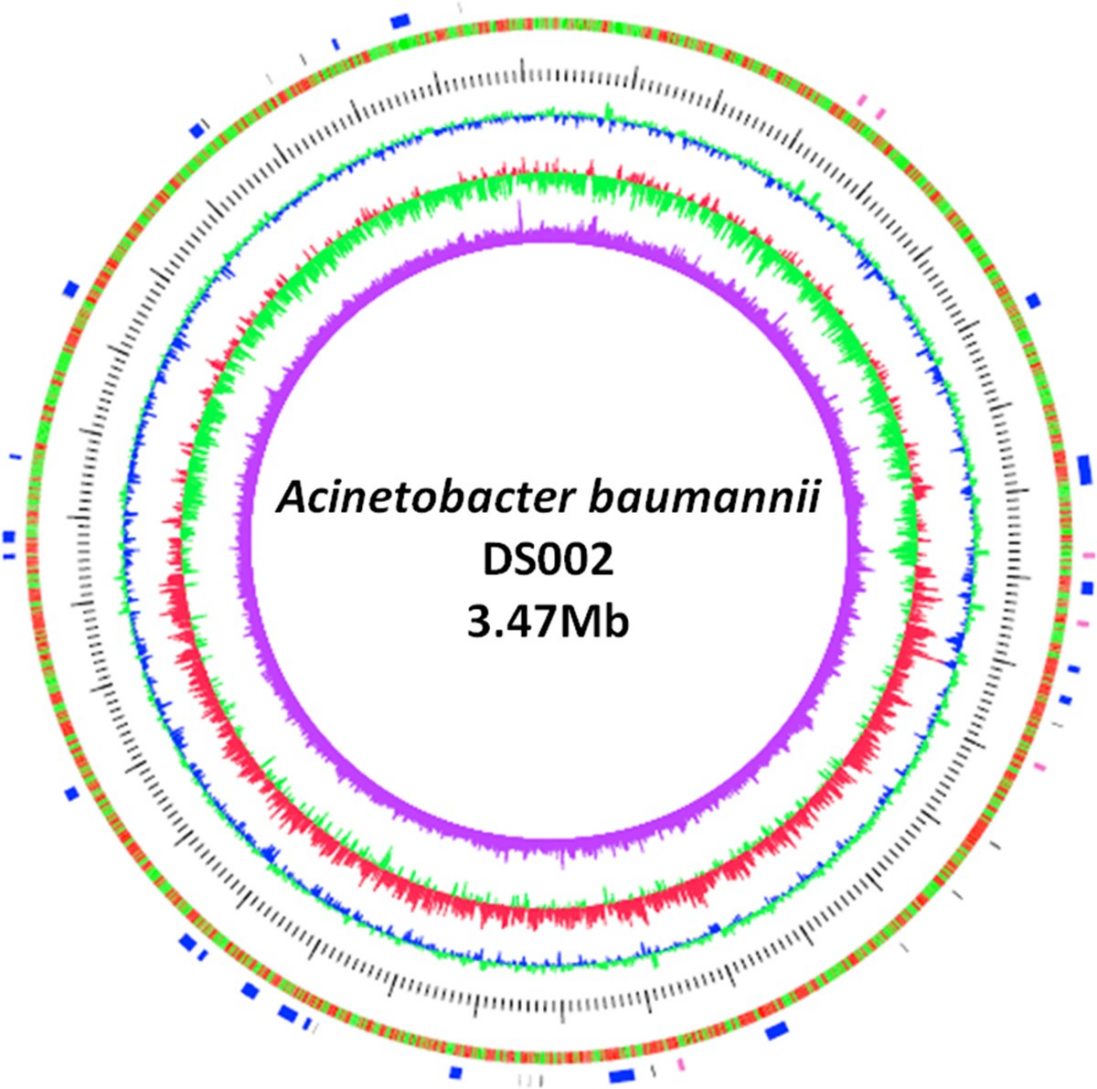
Circular map of the chromosome. Circle 1 (outer to inner) represents the total 3251 CDS in the genome. Genes present on the positive and negative strands are depicted in red and green colour, respectively. Circles 3, 4 and 5 represent GC content, GC skew, and dinucleotide bias, respectively. The tRNA (black) and rRNA (red) coding genes and the genomic islands (blue) are shown in the outermost discontinuous circle.

**Fig 2:**
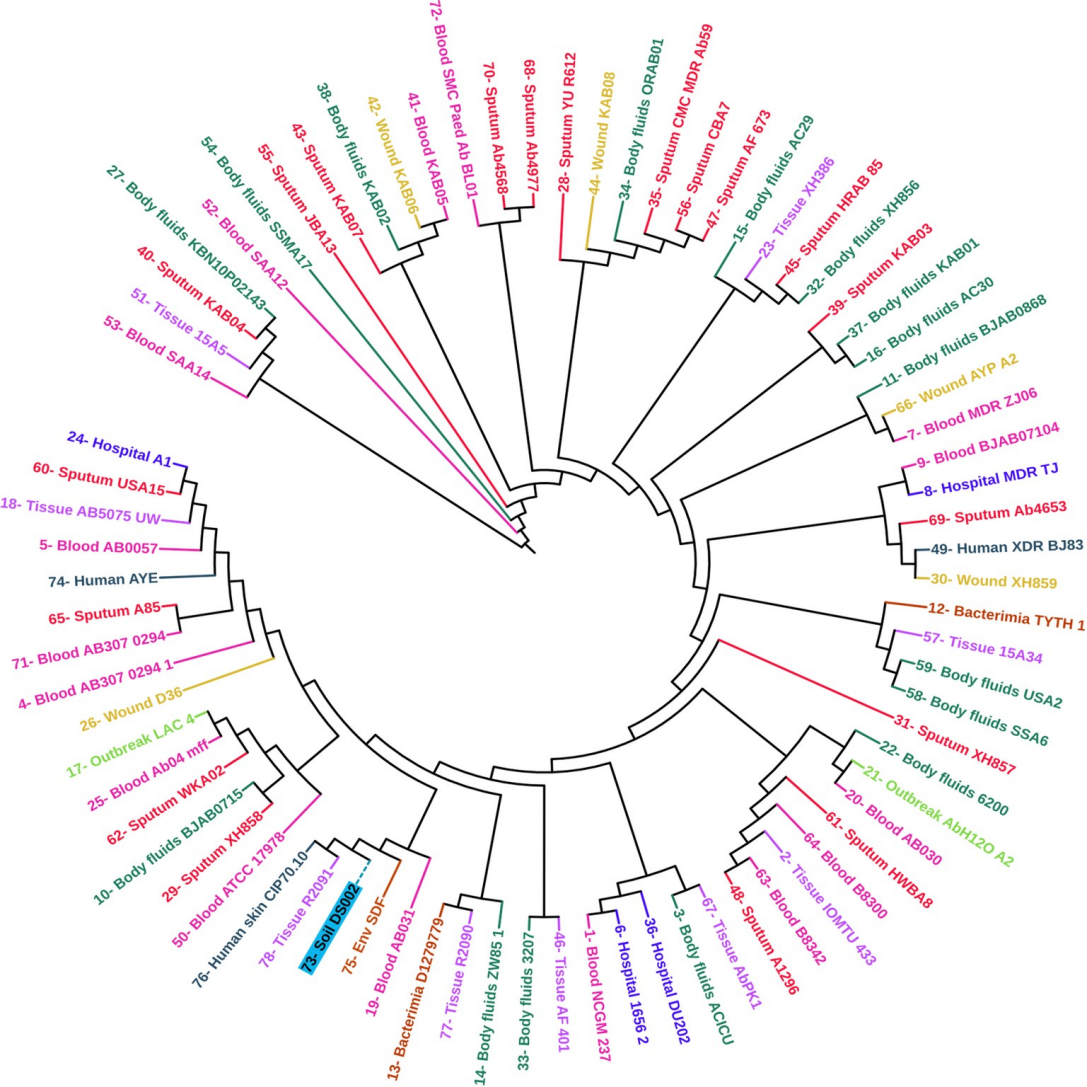
Phylogenetic tree. Core genome-derived phylogenetic tree of 78 *A*. *baumannii* strains. The position of DS002 in the phylogenetic tree is highlighted with a blue background and dotted clade line.

The *de novo* assembly of the PacBio raw reads using the Canu-HISEA pipeline generated 8 completely circularized contigs (S1 Table). The circularized length reflects the length after removing the overlapping portion. The largest assembled contig corresponds to the bacterial chromosome with a circular length of 3,430,798 bps. The remaining contigs correspond to the plasmid repertoire of the organism. The whole genome sequence of *A*. *baumannii* DS002 is available in the NCBI database (Genome Submission-ID: SUB3752749, BioSample submission ID: SUB3749011, BioProject submission ID: SUB3749009, accession number: CP027704.1). The Prokka annotation pipeline identified 18 rRNA, 72 tRNA, 1 tmRNA and a total of 3,569 protein-coding genes on the main chromosome of DS002, with a genomic GC content of 39.6%, which complies with the already reported genomic data of *A*. *baumannii* strains. The position of the coding genes on positive and negative strands, the GC skew and dinucleotide bias ratio for DS002 is shown in the circular map of the genome (Fig 1). The single copy orthologous genes (BUSCOs) indicate evolutionarily conserved genes among organisms belonging to a taxonomic class. The BUSCO set of gammaproteobacteria contains a total of 452 conserved orthologous genes. Of these 452 genes, 422 were found in the DS002 genome, suggesting the genome is about 93.4% complete. According to the core and pan-phylogeny analysis, the DS002 strain clustered closely with *A*. *baumannii* SDF, a strain isolated from the body louse (*Pediculus humanus*) (Fig 2 and S1 Fig). Among the sequences of *A*. *baumannii*, the sequence of environmental strain SDF showed the lowest ANI at nearly 96% when compared to the rest of the genomes of *A*. *baumannii* strains (S2 Fig). Interestingly, the ANI score (~97.5%) of DS002 was close to the genomes of *A*. *baumannii* strains isolated from clinical sources. The UPGMA tree constructed using Euclidean distance (obtained from ANI value) showed that strain SDF is most distant and dissimilar when compared to other strains of *A*. *baumannii* (S3 Fig). Interestingly, the UPGMA tree analysis revealed that strain DS002 has the highest similarity to *A*. *baumannii* strains isolated from sputum. PCA plot generated to gain better visualization on diversity also showed no isolation source specific clustering of *A*. *baumannii* strains. Interestingly environmental isolates DS002 and SDF were found as outliers in the PCA plot (S4 Fig).

### Pan-genome analysis

**Fig 3:**
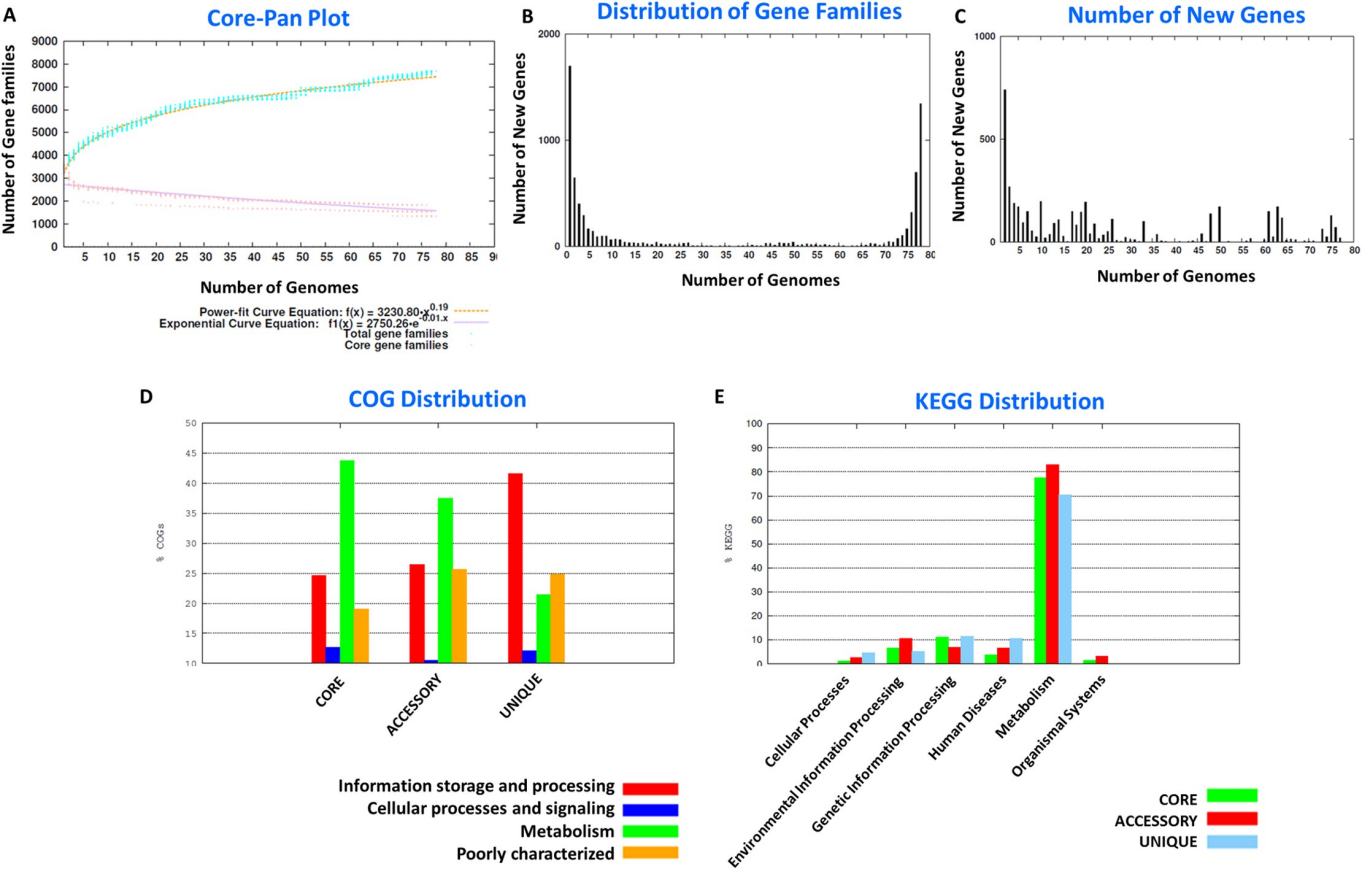
Pan genome analysis. Panel A indicates the pan and core genome curves. Panel B shows the frequency distribution of gene families within genomes. The number of new genes added to each genome is depicted in panel C. The COG and KEGG distribution of the representative proteins in the core, accessory, and unique genome are shown in panels D, and E respectively.

The *A*. *baumannii* complex forms the largest species group within the *Acinetobacter* genus. At present, there are about 135 complete genome sequences for the *A*. *baumannii* strains isolated from a variety of ecological niches. The primary aim of the study is to identify unique and accessory genes in the genome of *A*. *baumannii* species that might contribute to their exceptional survival strategies under diverse environmental conditions. We have therefore taken only 78 genomes for which the source of isolation is well documented (Table 1). Initially, we created a pan-genome pool by taking the sequences of all genes found in these 78 genomes (281,137 sequences) and segregated them as the core, accessory, and unique gene families. The pan-versus-core gene plot clearly indicates that the pan-genome window is still open for expansion in the case of *A*. *baumannii* (Fig 3A) and with the addition of each new genome to the *A*. *baumannii* complex a change in the pan-genome profile can result. Distribution of gene families and new genes within the pan-genome of the *A*. *baumannii* complex are shown in Fig 3B and 3C. Representative protein sequences of the core (1344), accessory (4644), and unique (1695) genes were identified and their COG and KEGG identities established. Accessory genes, which includes those present in only a few genomes, intriguingly are involved in cellular metabolism, emphasizing an ecological niche-specific change in the genome. Moreover, the genes involved in information storage and processing activities of the cell were all identified in the unique genes of the pan-genome (Fig 3D & 3E). The details of core, accessory and unique genes present in each of the 78 genomes are shown in the S2 Table. After segregation of pan-genome into the core, accessory, and unique genes, we specifically analyzed unique genes from the eleven representative strains to determine whether it is possible to identify a particular feature or features of the genetic composition that might contribute to niche-specific survival of *A*. *baumannii* strains.

### Unique genome content

In the unique genome content, we noticed genes that contribute to carbon catabolism, virulence, and drug resistance. As the analysis of the unique genes from all 78 genomes is challenging, we restricted our analysis to eleven representative genomes, taking one representative from each source of isolation with the highest number of unique genes. While analyzing the unique genes we found *kgdL* in the unique gene pool of *A*. *baumannii* 6200, isolated from the body fluid, which codes for the large subunit of membrane-bound gluconate 2-dehydrogenase [41]. Similarly, in *A*. *baumannii* HWBA8, a strain isolated from sputum, a membrane-bound PQQ-dependent dehydrogenase was identified as part of its unique genome. These membrane-bound dehydrogenases are linked to the cytoplasmic respiratory chain through their unique N-terminal domains. The PQQ-dependent dehydrogenases play a role in incomplete oxidation of monosaccharides leading to accumulation of breakdown products in the culture medium [41]. The physiological role of these membrane-bound dehydrogenases is still uncertain; however, results of several studies suggest that they provide additional energy-conserving systems, which aids the survival of the strain in environments with high energy demands [42–44].

### The *hrgA* gene exists in the unique genome of AB031

In blood isolate AB031, a *hrgA* gene was identified as one of the unique genes. The *hrgA* gene was initially identified in *Helicobacter pylori*, a well-known pathogen in the human gastrointestinal tract, as part of the restriction and modification system [45]. In *H*. *pylori* the *hrgA* gene has replaced the restriction component *hyp*IIIR locus. The existence of *hrgA* has been reported in nearly 33% of the sequenced *H*. *pylori* strains. Although the physiological or evolutionary significance of such gene replacement is unknown, a recent clinical study associated the *hrgA* gene with an increased incidence of gastric cancer [46]. The *hrgA* gene in *H*.*pylori* is present upstream of the *hypIIIM* gene encoding for the methylase component of the HypRM system. Since its existence is unique in AB031, we have analyzed the sequence regions 1000 bps upstream and downstream of the *hrgA* gene to determine whether *hrgA* also forms a part of such restriction and modification system in *Acinetobacter* sp. Interestingly, there was no indication from the DNA sequences of any association of *hrgA* with a modification system. Instead, we observed a gene coding for glycine tRNA synthetase subunit β and a gene coding for an uncharacterized transporter in the upstream and downstream regions of *hrgA*, respectively. Since the existence of *hrgA* is of clinical significance we searched all 3539 *A*. *baumannii* genome sequences to identify the *hrgA* gene. Our BLAST search revealed the presence of *hrgA* only in two genomes, namely *A*. *baumannii* 1297 (perirectal) and *A*. *baumannii* AB136 (blood). Both of them are clinical isolates and were isolated from human samples. Several studies have reported the occurrence of horizontal gene transfer between *A*. *baumannii* and bacteria belonging to other genera. It is conceivable that if *A*. *baumannii* frequently co-infects the host together with these *hrgA*-bearing pathogens, there is a possibility for *A*.*baumannii* to acquire *hrgA* from, for example, *H*. *pylori*, through HGT (41).

### The resistome is part of the unique gene pool in AYE

Several studies performed on the human pathogen AYE have established it as an XDR strain of *A*. *baumannii* complex. The genes conferring drug resistance, including *aadB*, encoding a 2-aminoglycoside nucleotidyltransferase, *bla*_*OXA-10*_ encoding beta-lactamase OXA-10 precursor, *dhfrI* encoding for dihydrofolate reductase type 1, *dhfrA10* coding for dihydrofolate reductase type A10, and *veb-1 en*coding the extended-spectrum beta-lactamase were identified in the unique genome of the AYE strain.

### Rut pathway encoding genes are in the unique genome of B8342

The unique genome of blood isolate B8342 contains genes involved in pyrimidine degradation via the Rut (pyrimidine utilization) pathway. The *rutABCDEFG* operon gene products facilitate the use of pyrimidines as a sole nitrogen source. In *E*. *coli*, the *rut* operon is part of NtrC regulon [47] and is highly expressed under nitrogen-limiting conditions [48]. The *rutA* gene codes for pyrimidine oxygenase and is involved in ring cleavage of pyrimidines, thereby producing the toxic ureidoacrylate peracid. The *rutF* gene is a flavin reductase involved in the regeneration of the flavin mononucleotide cofactor, while *rutB* encodes an amidohydrolase and is involved in the further breakdown of peracid *in vitro*. The *rutBCD* genes are assumed to catalyze the breakdown of ureidoacrylate peracid *in vivo*, subsequently releasing toxic malonic semialdehyde [49]. In *E*. *coli*, two genes, *rutE*, and *ydfG* have been reported to convert malonic semialdehyde into 3-hydroxypropionate [50]. Apparently, the Rut pathway is functional only at low, or at room temperature, and not at 37°C [51], as the toxic malonic semialdehyde cannot be converted into 3-hydroxypropionate at 37°C [48]. The physiological concentration of pyrimidines in blood plasma and other body fluids is around 0.4–0.6 μM, which is considerably lower than the intracellular concentrations of 0.5–3 mM [52]. If a pathogen acquires the capacity to utilize pyrimidines as a source of nitrogen, it certainly would be advantageous to blood isolates like *A*. *baumannii* B8342. The Rut pathway has also been identified among soil proteobacteria and is suggested to have a role in utilizing pyrimidines generated from decaying plant material as a source of nitrogen [49]. Identification of the Rut pathway in a pathogenic strain signifies its importance in the survival of organisms with pathogenic lifestyles. In contrast to the clinical isolates, the major portion of the unique genome of non-clinical isolate SDF comprises hypothetical genes. Interestingly, its unique genome codes for putative hemaglutinin/hemolysin-related proteins [11]. The presence of such genes in the SDF strain might help its survival as an ectoparasite by feeding on human blood.

### Comparison of carbon catabolome

**Fig 4:**
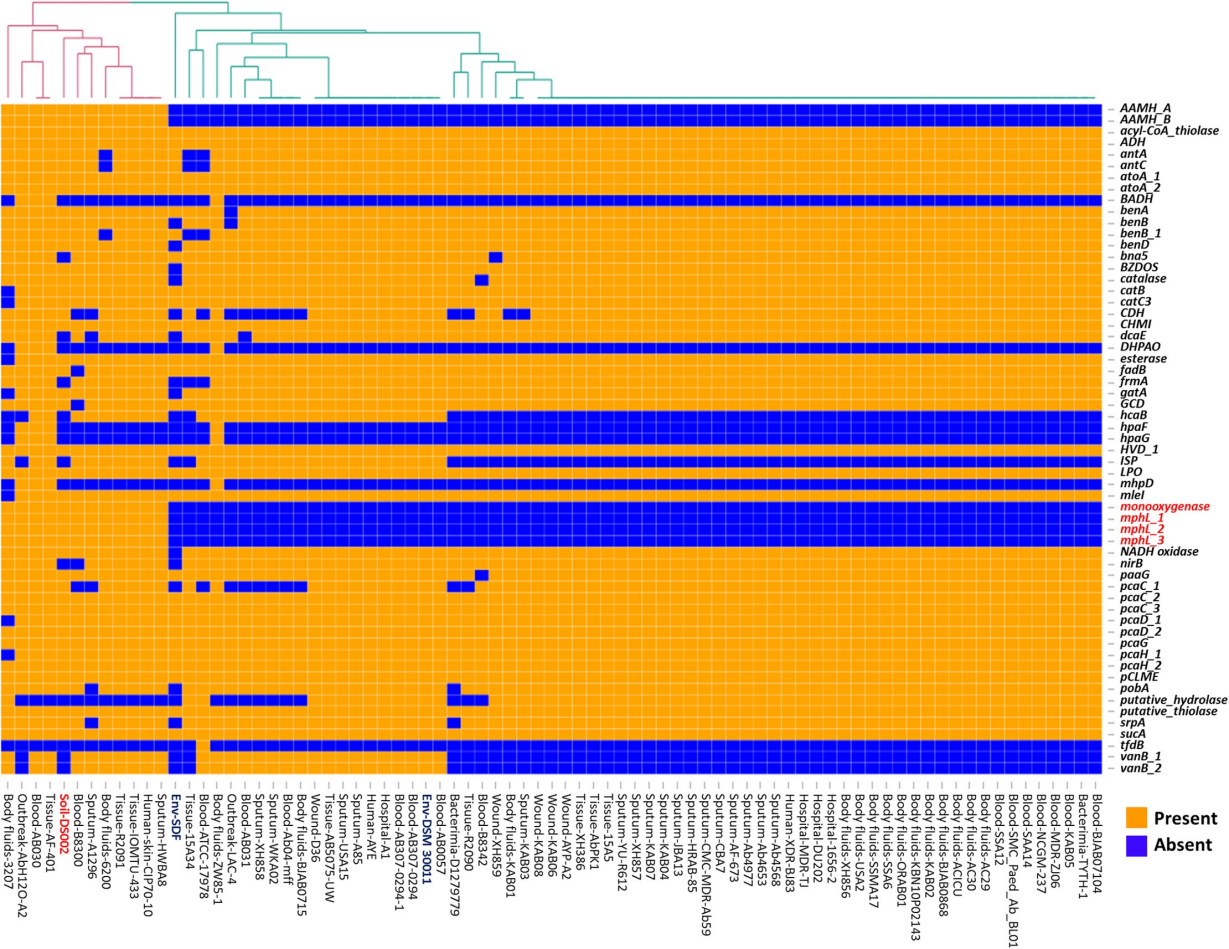
Heat map showing LS-BSR analysis of genes involved in carbon catabolism. Genes extensively discussed in the text as well as the strain DS002 are highlighted in red font.

Most of the *A*. *baumannii* strains used in the study contained similar carbon metabolic pathways, and show no preference for a specific carbon source (Fig 4). However, the soil isolate includes genes coding for phenol 2-monooxygenase (*pheA*) and phenol hydroxylase *(mphL)* enzymes. Interestingly, these genes were absent in most of the clinical isolates. Phenol hydroxylase is a multicomponent monooxygenase that cleaves the aromatic ring and comprises three enzymatic components including a reductase, an oxygenase, and a regulatory component [53]. The oxygenase component has a dinuclear iron center at its active site, in which an oxygen atom is complexed by two iron ions. The reductase component transfers electrons to the dinuclear iron center facilitating the initial hydroxylation and subsequent detoxification of recalcitrant aromatic compounds such as phenol, benzene, toluene, xylene, and methyl or chlorophenols, to generate catechol. Catechol is subsequently degraded either by *ortho*- (*Acinetobacter sps*) or *meta-*cleavage (*Pseudomonas spp*) pathways [54, 55] enabling the cells to use these rare and recalcitrant carbon compounds as a source of carbon and energy.

Monooxygenase-encoding *pheA* genes have been identified in a number of soil microbes including yeast *Trichosporon cutaneum*,[56], *Pseudomonas pickettii* PKO1[57], *Bacillus stearothermophilus* BR219 [58], and some species of *Acinetobacter* [59]. A recent study has also shown the presence of a gene coding for phenol hydroxylase in pathogenic strains of *A*. *baumannii*. Existence of catabolic potential for phenol in pathogenic *A*. *baumannii* strains is a matter of huge concern as phenolic substances are used as disinfectants in hospitals [60]. Since strain DS002 contains the genetic capability for metabolizing phenolic substances we tested if it can grow using phenol as the sole source of carbon. Consistent with the genetic information, DS002 was able to grow in minimal salt medium containing 2mM phenol as the sole source of carbon. However, *A*. *baumannii* strain AYE in which no *pheA* homologues were identified failed to grow under similar culture conditions (S5 Fig). Our study has also revealed the presence of genes involved in phenol degradation in *A*. *baumannii* strains isolated from different body fluids (Fig 4) and this feature of these strains poses a major challenge in the use of phenol-based disinfectants in hospital environments.

### Virulome of *A*. *baumannii* strains

**Fig 5:**
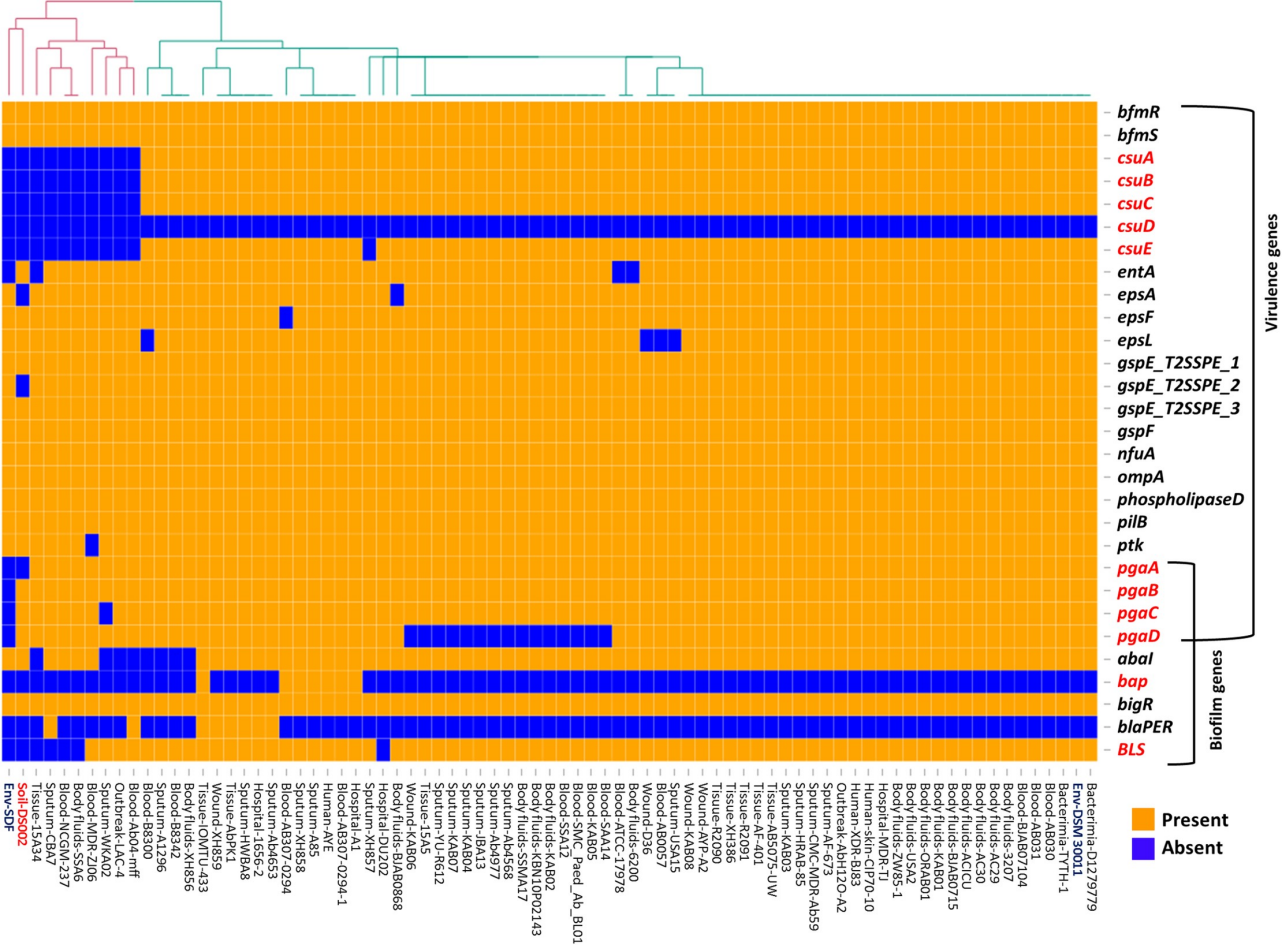
Heat map showing LS-BSR analysis of genes involved in virulence. Genes extensively discussed in the text as well as the strain DS002 are highlighted in red font.

Analysis of the virulome of the *A*. *baumannii* strains revealed the presence of similar virulence genes in most of the clinical isolates, suggesting the pathogenic nature of the majority of stains within the *A*. *baumannii* complex (Fig 5). Interestingly, a significant variation was observed with respect to the biofilm-associated genes. In the environmental isolates, the operon coding the csu pili chaperone-usher assembly system, which is known to contribute to biofilm formation [61], is only present in pathogenic *A*. *baumannii* strains and is absent in the genome of the soil isolate DS002 and the SDF strain (Fig 5). In addition to the *csu* operon, the biofilm-associated protein (*bap*) and blue light-sensing (*bls*A) gene, whose roles in biofilm formation have been established, were found in all pathogenic strains of *A*. *baumannii* [62–65]. None of these biofilm-associated virulence genes was observed in soil isolate DS002 and the SDF strain isolated from the body louse [11]. Interestingly, strain DS002 also carries the *pgaABCD* operon reported to be essential for the production, modification, and export of poly-β-1,6-N-acetyl-D-glucosamine (β-1,6-GlcNAc; PGA). The roles of *pgaC* and *pgaD* genes in the biosynthesis of poly-β-1,6-N-acetyl-D-glucosamine (PGA) is well established. The *pgaB* gene encodes an N-deacetylase that converts 15–20% of GlcNAc to glucosamine and *pgaA* codes for a membrane porin required for PGA export [66–68]. The absence of *pgaA* in DS002 might weaken the polysaccharide matrix necessary for biofilm formation. Similarly, the entire *pgaABCD* operon is absent the genome of the SDF strain (Fig 5). The role of PNAG in biofilm-mediated virulence has been previously reported in almost 30 clinical isolates of *Acinetobacter baumannii* [69]. In agreement with the genetic evidence, the multidrug-resistant AYE strain, which has the genetic repertoire for biofilm production, was significantly better at forming a biofilm compared with the soil isolate DS002, with no difference observed in their growth (S6 Fig).

### Comparison of the resistome

**Fig 6:**
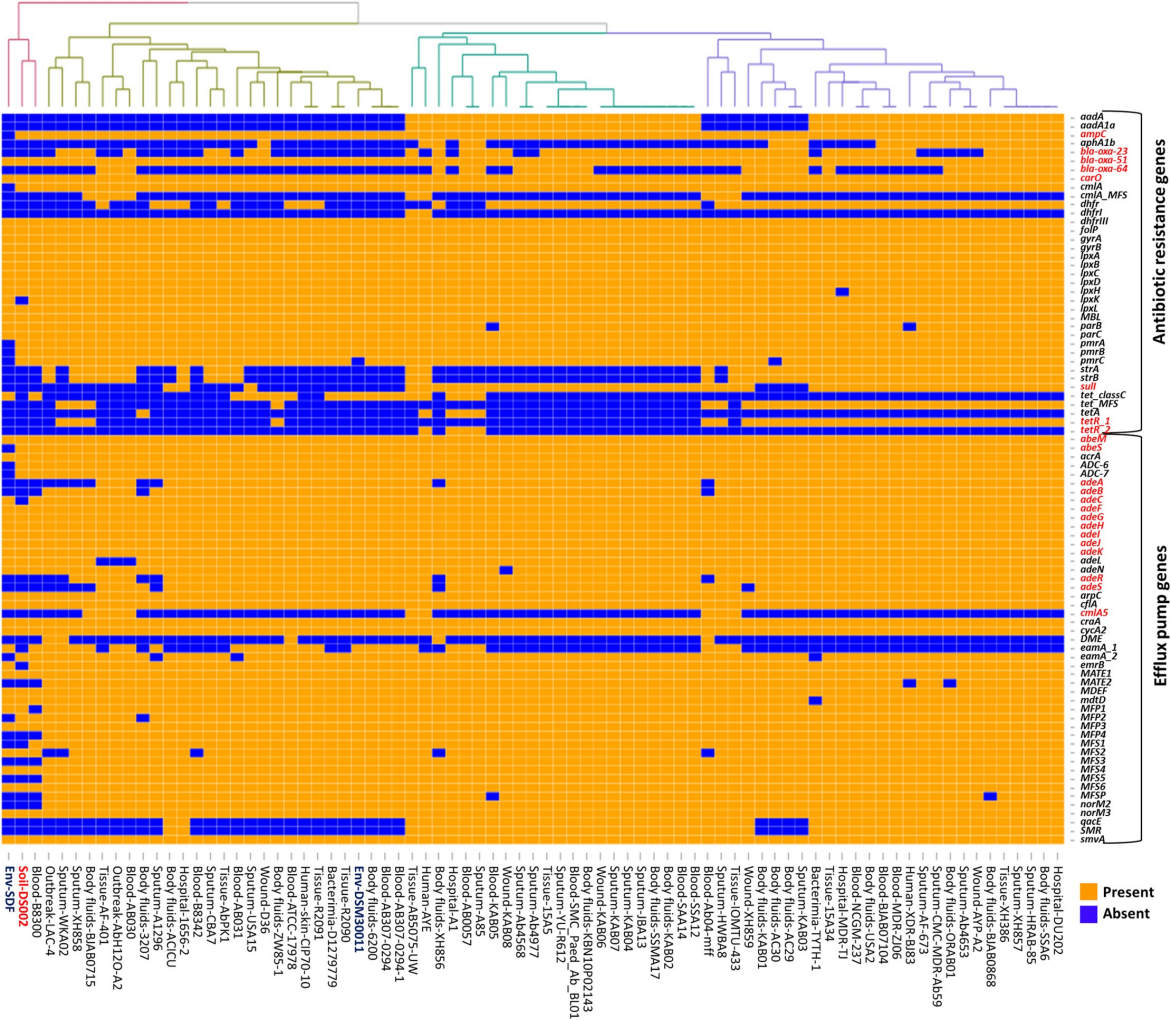
Heat map showing LS-BSR analysis of genes involved in resistance. Genes extensively discussed in the text as well as the strain DS002 are highlighted in red font.

Strains belonging to the *A*. *baumannii* complex show intrinsic β-lactamase activity due to the existence of β-lactamases like the cephalosporinase AmpC [17]. These genes, when associated with the IsAba1-type insertion element, confer resistance to 3^rd^ generation cephalosporins [17]. The weak oxacillinase activity of *A*. *baumannii* strains is attributed to the presence of bla_OXA-51/69_ type oxacillinases [70]. An increased oxacillinase activity, which confers resistance to imipenem, is associated with the presence of *IsAba1*-type insertion elements [71, 72]. Most of the strains considered in our study, irrespective of their source of isolation, have shown the presence of chromosomally located bla_OXA-51_-like genes. A subset of oxacillinases is known to exhibit weak carbapenemase activity when compared to the metallo-β-lactamases and hence are grouped in the carbapenem-hydrolyzing class-D β-lactamases [17]. In *A*. *baumannii*, different types of Carbapenem-Hydrolyzing Class D β-Lactamases (CHDLs) are observed, which include, OXA-23-like, OXA-40/24-like, OXA-58-like [73], OXA-143-like [74], and OXA-235-like [75]. Significantly, the present study revealed the existence of an OXA-23-like CHDL gene as part of the unique genome in strain IOMTU 433, due to its presence as part of mobile genetic elements [76, 77]. The presence of both intrinsic *bla*_*OXA*_64 (51-like) and acquired *bla*_*OXA*_23 genes was observed in *A*. *baumannii* IOMTU 433. Carbapenem-associated outer membrane protein (CarO) is a membrane porin that facilitates uptake of imipenem due to the presence of an imipenem-binding site in the protein (61). Its absence in the genome of DS002 strain, but the presence of *ampC*, suggests possible weak carbapenemase activity exhibited by the strain (Fig 6).

Genome sequences of certain strains have shown the presence of genes that code for broad-spectrum β-lactamases, such as cephalosporinase-*blaADC*, and *TEM-1* β-lactamases. Tissue isolate IOMTU 433 also possesses an *NDM-1*metallo-β- lactamase gene, making the strain resistant to all known classes of β-lactams [78]. The extremely drug-resistant human isolate of *A*. *baumannii* AYE was observed to contain *veb-1* (Vietnamese extended-spectrum beta-lactamase) genes, which provide resistance towards extended-spectrum cephalosporins and aztreonam.

In addition to β-lactamases, pathogenic strains were also found to express aminoglycoside-modifying enzymes conferring resistance to aminoglycoside antibiotics. All three types of aminoglycoside-modifying enzymes including i) N-acetyltransferases (AAC), which catalyze acetyl CoA-dependent acetylation of an amino group, 2) O-adenyltransferases (ANT) catalyzing ATP-dependent adenylation of hydroxyl group, and 3) O-phosphotransferases (APH) that catalyze ATP-dependent phosphorylation of a hydroxyl group on the aminoglycoside antibiotics [79], are found in the genome of infectious strains, leading to their successful emergence as MDR pathogens (Fig 6). Furthermore, the *sul1* gene has been identified in the genome of strains AYP-A2 and AYE, indicating their potential resistance to sulfonamides. However, none of these genes was identified in the genome of soil isolate DS002, probably owing to the strain’s limited exposure to such classes of antibiotics in the soil environment.

Efflux-mediated resistance in *A*. *baumannii* strains is well established, and it contributes significantly to MDR. The acinetobacter drug efflux (ade) pumps comprise the first Resistance-Nodulation-Division (RND) efflux pumps to be identified among the clinical isolates of *Acinetobacter* but is not present in environmental isolates. In accordance with previous reports, the environmental isolate DS002 lacks the *adeABC* RND efflux pump and the corresponding two-component regulatory system, *adeRS*, which contribute to resistance towards aminoglycosides, tetracyclines, erythromycin, trimethoprim, fluoroquinolones, some beta-lactams, and tigecycline [80–82]. However, it contains other RND efflux pumps such as *adeIJK* and *adeFGH*, which are involved in the efflux of ticarcillin, cephalosporins, aztreonam, fluoroquinolones, tetracyclines, tigecycline, lincosamides, rifampin, chloramphenicol, cotrimoxazole, novobiocin, and fusidic acid (Fig 6). Non-RND efflux pumps, such as *abeS* and *abeM*, were also found in the genome of DS002. All the representative pathogenic strains considered in our study were found to code for the *adeABC* efflux pumps. In addition to these, the AYP-A2 and AYE strains encoded Tet and CmlA efflux pumps, accounting for resistance to tetracycline and chloramphenicol.

In summary, the genome of strains belonging to *A*. *baumannii* complex contains numerous resistance genes and efflux pumps that are either characteristic of *A*. *baumannii* species and that have been presumably acquired, resulting in the drug-resistant nature of *A*. *baumannii* strains.

### Diversity in iron uptake mechanism

**Fig 7:**
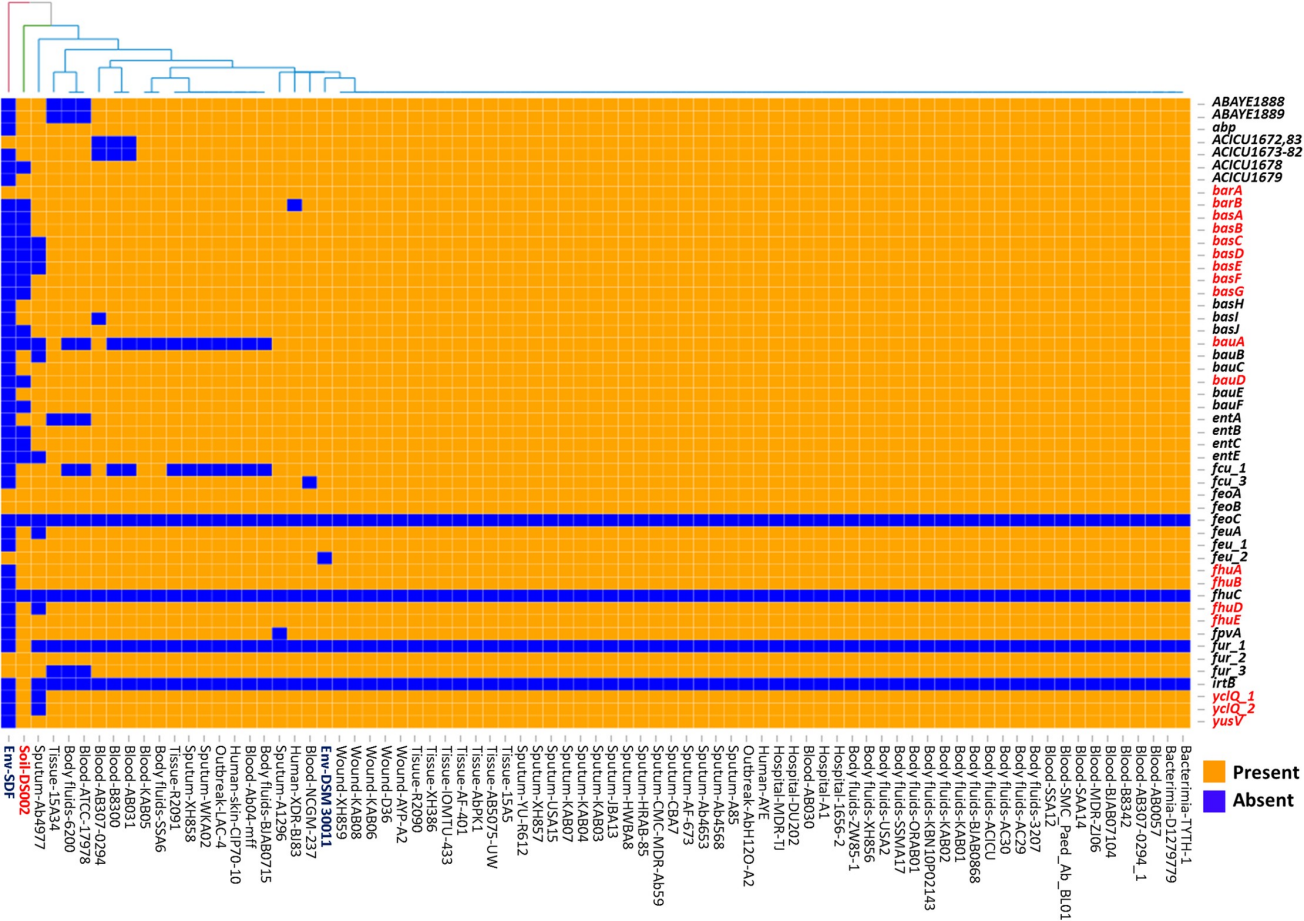
Heat map showing LS-BSR analysis of genes involved in iron acquisition. Genes extensively discussed in the text as well as the strain DS002 are highlighted in red font.

**Fig 8:**
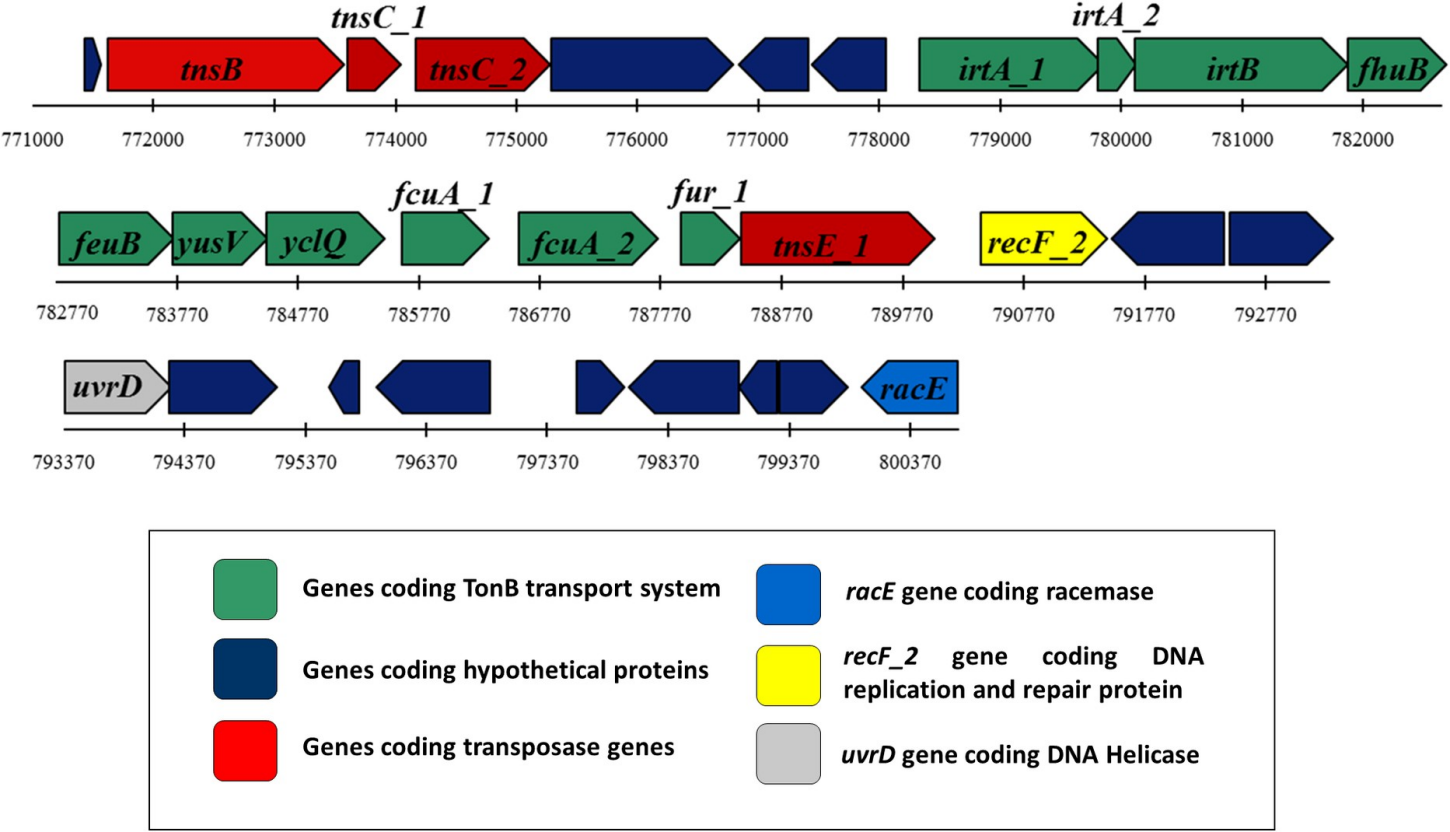
Genomic Island. The genetic map indicates the genomic island (30Kb) identified in the genome of DS002 with putative iron acquisition machinery. Genes coding a TonB transport system and hypothetical proteins are shown with dark green and blue arrows, respectively. The genetic map also indicates the presence of *tnsB*, *tnsC*_1, *tnsC*_2, *tnsE*_1 encoding a transposase (red), along with *uvrD* (grey), *racE* (light blue) and *recF2* (yellow) encoding DNA helicase, racemase, and a DNA replication/repair protein, respectively.

Iron acquisition is a major challenge faced by soil bacteria due to the insolubility of Fe (III) in aerobic habitats. Over the period of evolution, bacteria have developed several mechanisms to acquire Fe (III) from the environment. Siderophores are iron-scavenging molecules that are synthesized and released by bacteria for iron uptake. In the genome sequences of *Acinetobacter*, five major siderophore biosynthetic clusters were reported [83, 84]. Prominent among these is acinetobactin; its biosynthetic genes are found in all genome sequences of *A*. *baumannii* except in the non-clinical isolate *A*. *baumannii* SDF strain [24, 84]. The genome of soil isolate DS002 lacked most of the acinetobactin biosynthetic genes (*basABCDEFGJ*) except for *basH* and *basI* or the genes involved in acinetobactin uptake (*bauAD*) except *bauB*, *bauC* and *bauE*, and release (*barAB*) (Fig 7). Similarly, biosynthesis of the siderophore fimsbactin was specifically identified in *A*. *baumannii* ATCC 17978 and ADP1 [85]. This siderophore biosynthetic gene cluster is absent in the DS002 strain. However, the genome of DS002 appears to have all genes involved in the biosynthesis of the siderophore identified in *A*. *baumannii* ACICU (gp1672-1683), except the gene that codes for demethylmenaquinone methyltransferase. As this type of iron acquisition mechanism is poorly studied, little information pertaining to the gene products is available in the literature. Some of the genome sequences of *A*. *baumannii* complex reveal the presence of genes coding for a catecholate siderophore (*A*. *baumannii* ABAYE1888-1889), encoding isochorismatase and a 2, 3-dihydro-2,3-hydroxybenzoate dehydrogenase [86]. These were identified in the genome of DS002. These enzymes produce 2,3-dihydroxybenzoate, an iron-binding molecule, which also serves as a precursor for the synthesis of complex siderophores [86]. The siderophore-mediated uptake of iron by Gram-negative bacteria is facilitated by the presence of TonB-dependent transport systems comprising an outer membrane receptor, a periplasmic binding protein, and an inner membrane permease. Several iron-responsive *ton*B-dependent receptors involved in cognate siderophore recognition and uptake have been identified in *Acinetobacter*. Genes coding for *fhuA*, an outer membrane ferrichrome receptor, *fhuB*, an inner membrane permease, and *fhuD*, encoding a periplasmic binding protein, were identified in the genome of DS002, indicating the probable uptake of a ferrichrome-type siderophore. Interestingly, most siderophore-related genes were found on a large GI (771Kb - 800Kb, ~30Kb) predicted in the genome of *A*. *baumannii* DS002 (Fig 8). This island contains 30 different ORFs, of which four encode putative transposases, 13 hypothetical genes, and the remaining 13 ORFs appear to be involved in iron acquisition. The genes include *fhuB*, a permease involved in ferrichrome uptake, *feuB*, a permease involved in enterochelin uptake and *fcuA*, a receptor for ferric citrate uptake, along with the *fur* gene, which is the master transcriptional regulator of the iron-responsive genes. The genes *yus*V and *ycl*Q, also found in this island, encode putative siderophore-transport system ATP-binding proteins.

### Genomic islands (GIs)

After identifying a GI including genes encoding the iron uptake machinery in DS002, we then looked for GIs in the eleven representative strains. Most of the *A*. *baumannii* strains contain on an average 30–40 GIs per genome, indicating a high degree of horizontal mobility of DNA among the strains of the *A*. *baumannii* complex. In pathogenic *A*. *baumannii* strains, the genes associated with the predicted GIs encode proteins and enzymes involved in either aromatic degradation, formaldehyde detoxification, the type I secretion pathway for protein translocation, restriction-modification systems, amino acid biosynthesis, competence proteins, exoribonuclease VII, or heavy metal resistance (mercury, copper, and arsenic). All predicted GIs can be considered as contributing to general fitness and facilitate survival of the respective strains under unfavorable environmental conditions. The genes contributing to resistance to gentamicin, sulfonamides, β-lactams, streptomycin, and their respective efflux pumps were found on GIs in drug-resistant strains such as AYE, HWBA8, AYP-A2, which were isolated from human, sputum and wound sites, respectively. Interestingly, the majority of GIs in pathogenic strains was found to code for bacteriophage-related genes with a putative function in phage assembly and maintenance (at least 8–59 copies of phage genes). Contrary to the GI landscape in pathogenic strains, the GIs in the soil isolate DS002 did not code for any of these genes, except for copper resistance. Additionally, it contained two copies of prophage integrase genes, which were not seen in other strains, and an island that encoded the ferripyoverdine receptor (*fpv*A), that is known to be involved in the uptake of the pyoverdine siderophore [87]. In both DS002 and the AYP-A2 strains, one of the islands was found to encode all the NADH: quinone oxidoreductase subunits, which play a key role in respiration.

## Discussion

Isolation of MDR strains of *A*. *baumannii* has gradually increased ever since they were first identified during an outbreak at a hospital in New York City [88]. In the 1970s, most of the strains were sensitive to well-known antibiotics. Meanwhile, during the span of forty years nearly 70% of the clinical isolates have acquired MDR status [2]. Carbapenems, once considered to be the linchpin against MDR *A*. *baumannii* strains were found to be ineffective in controlling MDR strains of *A*. *baumannii*. Such a rapid increase in drug resistance requires deeper insights into the correlation between the evolution of *A*. *baumannii* and their adaptation to a pathogenic lifestyle.

The comparative genomics performed between the soil isolate DS002 and strains isolated from hospital settings and from body fluids, clearly suggests the existence of unique gene content that contributes to the niche-specific survival of the strains.

The ability of *A*. *baumannii* strains to colonize humans depends on the initial establishment of contact with an epithelial cell of the body. The outer membrane porin OmpA is shown to facilitate such contact with the host cells [89]. Once contact is established, the ability of the bacterium to survive in the host depends on its ability to form a protective biofilm. Therefore, the comparative genomic study clearly indicated the existence of the complete genetic machinery for biofilm formation in all strains adapted to a pathogenic lifestyle. However, in strains adapted to survive in soil or water such a genetic machinery is either completely or partially absent. Experimental evidence generated using DS002 and AYE strains have confirmed these findings based on genomic analyses. The AYE strain showed a better biofilm formation ability than DS002 and in contrast, the strains adapted to a pathogenic lifestyle have lost genes required for degradation of rare and/or recalcitrant carbon compounds, such as phenol, as a sole source of carbon and energy. The catabolome required for thriving on phenol or benzoate was only found in environmental isolates. These catabolic traits are non-essential for the strains adapted to a pathogenic life style because they will not be confronted with such chemicals. Strain DS002 was isolated from insecticides-polluted agricultural soils and most commonly used insecticides generate phenolic compounds during the course of their biodegradation [90]. Therefore, they can be a rich carbon source for soil-dwelling bacteria, and hence strain DS002 acquired and retained the catabolome for metabolizing phenol, but the pathogenic strain AYE did not. The ability to degrade phenol as part of a unique genome content in environmental isolates has to be viewed with caution as most of the disinfectants used in hospital settings meanwhile also contain phenolic substances. If *A*. *baumannii* strains found in hospitals/intensive care units acquire the capacity to degrade phenol via horizontal gene transfer new disinfectants will need to be developed to remove such strains from nosocomial environments.

Interestingly, when the Rut pathway is considered, certain human isolates retained the corresponding genes. This may be due to the continual replenishment of the nucleotide pool from dead tissue or decaying plant material in the ecological niche and the expression of the Rut pathway is advantageous for the survival of the *A*. *baumannii* strains surviving in that habitat.

Irrespective of their lifestyle, acquisition of iron is a major nutritional challenge to all *A*. *baumannii* strains. All of them have a well-established transport system for importing iron bound to iron-chelating siderophores. Most of the bacteria possess an endogenous ability to synthesize their own siderophores. However, certain bacteria have efficient siderophore transport systems despite not having an innate ability to synthesize siderophores. In such cases, they presumably scavenge iron bound to siderophores made by other bacteria. The xenosiderophore transport system exists in DS002 on a GI, which certainly helps to compensate for strain’s inability to make its own siderophores (Fig 8).

Likewise, significant niche-specific genetic differences are seen with respect to drug resistance. The genes coding for all three types of aminoglycoside-modifying enzymes is absent in the genome of both DS002 and SDF strains. Similarly, the *adeABC* operon encoding RND efflux pumps and the corresponding two-component regulatory system, *adeRS*, are absent in soil isolates. Therefore, strain DS002 is sensitive to aminoglycosides, tetracyclines, erythromycin, trimethoprim, and fluoroquinolones (data not shown). However, the genome of DS002 indicates the presence of *adeIJK* and *adeFGH*RND efflux pumps along with other non-RND efflux pumps like a*beS* and *abeM*, suggesting that these potentially contribute to the resistance of the respective strain towards certain antibiotics like chloramphenicol, cotrimoxazole, novobiocin, and fusidic acid.

Interestingly, the virulome of *A*. *baumannii* is conserved in all *A*. *baumannii* strains. The presence of virulence factors, such as the BfmRS system, OmpA, phospholipase D in DS002, suggests a latently infectious property of the strain, irrespective of the source of its isolation. However, the soil isolates have lost the potential to infect humans due to a lack of biofilm formation ability. As the virulence factors are available in all *A*. *baumannii* strains, the soil-dwelling strains could, therefore, acquire the potential to colonize humans if they picked up genes encoding biofilm formation through HGT. Thus, the comparative genome analysis highlights not only apparent genetic differences found among *A*. *baumannii* strains but also the survival strategies being adopted by these *A*. *baumannii* strains.

## Conclusion

The comparative genome analysis highlighted the selective expansion of unique, niche-specific genome content in *A*. *baumannii*. The expanded unique genome content contribute to the strain’s adaptability to different ecological habitats. Our study clearly revealed the expansion of drug-resistance genes only in clinical isolates, as it confers a selective advantage for the survival of clinical isolates in an ecological niche that is frequently exposed to all kinds of antibiotics. Interestingly, no difference in the virolome of the different *A*. *baumannii* strains exists. However, the genetic makeup required for biofilm formation, an essential feature for colonization of a host, is only seen in clinical isolates. Because biofilm formation is not essential for the survival of soil isolates, the loss of critical genes involved in biofilm formation is frequently observed. Niche-specific genome expansion has also revealed an impact on carbon metabolism. The *pheA* found in unique genome content enables the survival of cells using phenol as the sole source of carbon, is only seen in strains that are frequently exposed to phenolic substances.

## Supporting information

S1 File Customized list of genes involved in virulence, resistance, iron acquisition, and carbon metabolism in *A. baumannii*. (XLSX)

S1 Fig Pan-genome derived phylogenetic tree of 78 *A. baumannii* strains. The position of DS002 in the phylogenetic tree is highlighted with a blue background and dotted clade line.
(TIF)

S2 Fig Heatmap showing Average Nucleotide Identity (ANI) for all 78 genomes. (TIF)

S3 Fig UPGMA tree drawn using ANI-based Euclidean distance. Strains with similar isolation source are shown with identical colors. The dotted blue line indicates the clade of soil isolate DS002 highlighted in blue.
(TIF)

S4 Fig PCA analysis of all genomes based on the LS-BSR output. Strains with similar isolation source are shown with identical colors.
(TIF)

S5 Fig Growth of DS002 (filled circle) and AYE (filled square) in minimal medium supplemented with 2mM phenol as the sole source of carbon. (TIF)

S6 Fig Panel A represents crystal violet staining of tubes used to grow AYE (I) and DS002 (II). OD_590_ values obtained for the ethanol extracts prepared from these two tubes indicating the extent of biofilm formation is shown in panel B. Panel C represents the bacterial growth as measured by OD_600_.
(TIF)

S1 Table Six circularized contigs obtained by *de novo* assembly. (DOCX)

S2 Table The distribution of core, accessory, unique and exclusively absent genes in all the 78 *A. baumannii* strains under study. (DOCX)
