# Supplementary figures and images for: Comparative genome analysis reveals niche-specific genome expansion in Acinetobacter baumannii strains
Source: PLoS One. 2019 Jun 13;14(6):e0218204. doi: 10.1371/journal.pone.0218204 (PMC6563999; doi:10.1371/journal.pone.0218204)

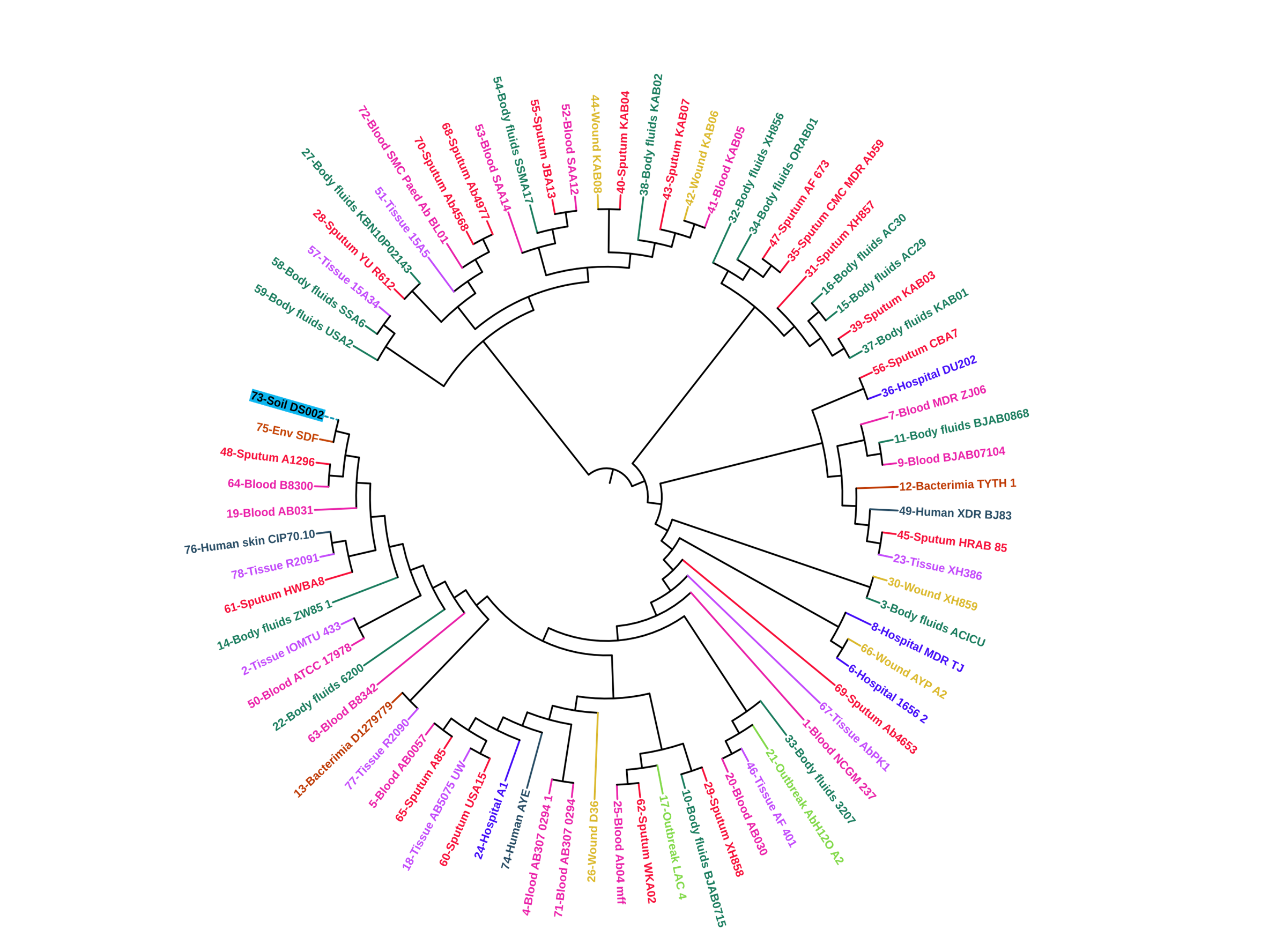

Supplement: S1 Fig — The position of DS002 in the phylogenetic tree is highlighted with a blue background and dotted clade line. (TIF) [file pone.0218204.s002.tif]

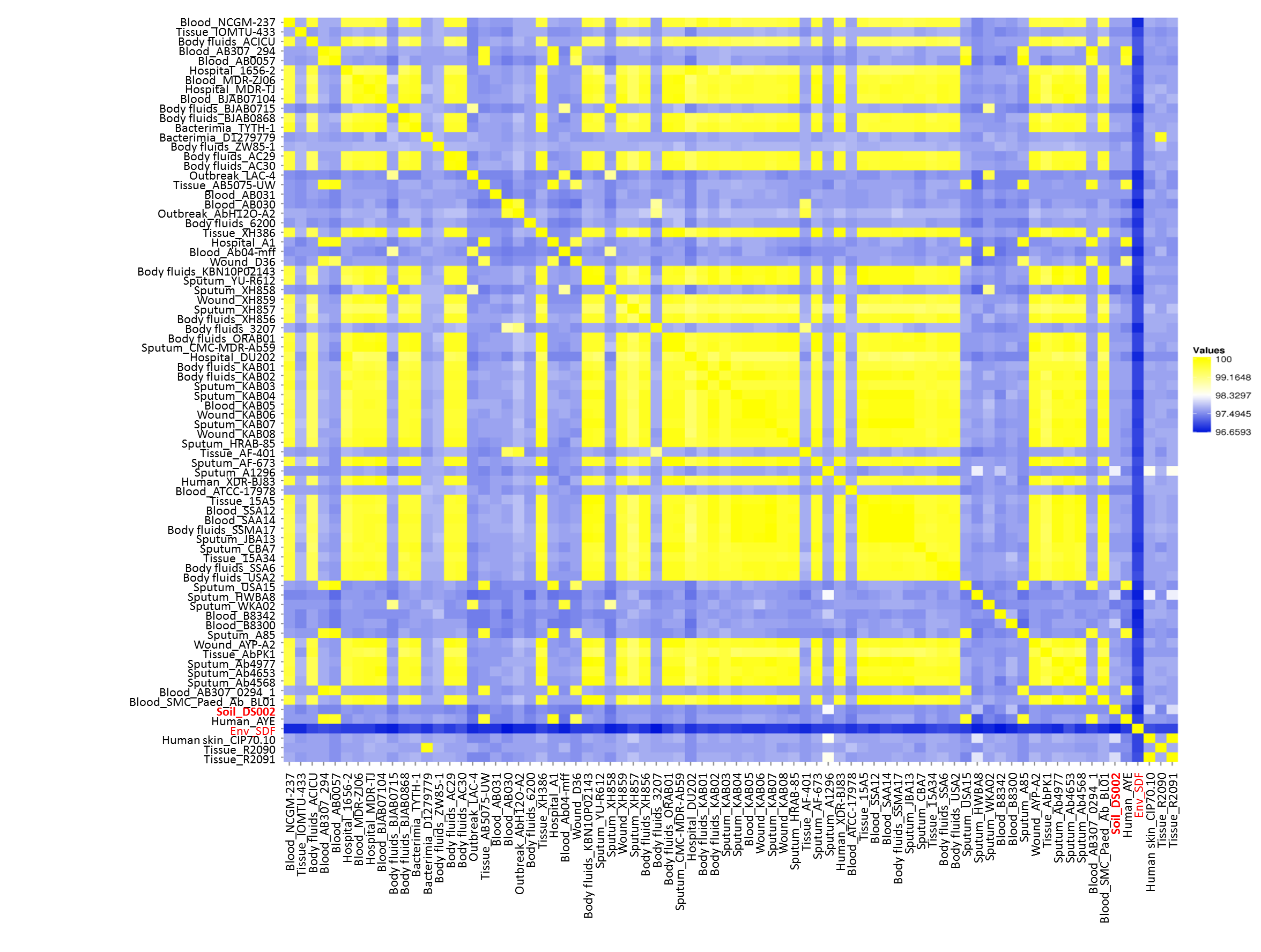

Supplement: S2 Fig — (TIF) [file pone.0218204.s003.tif]

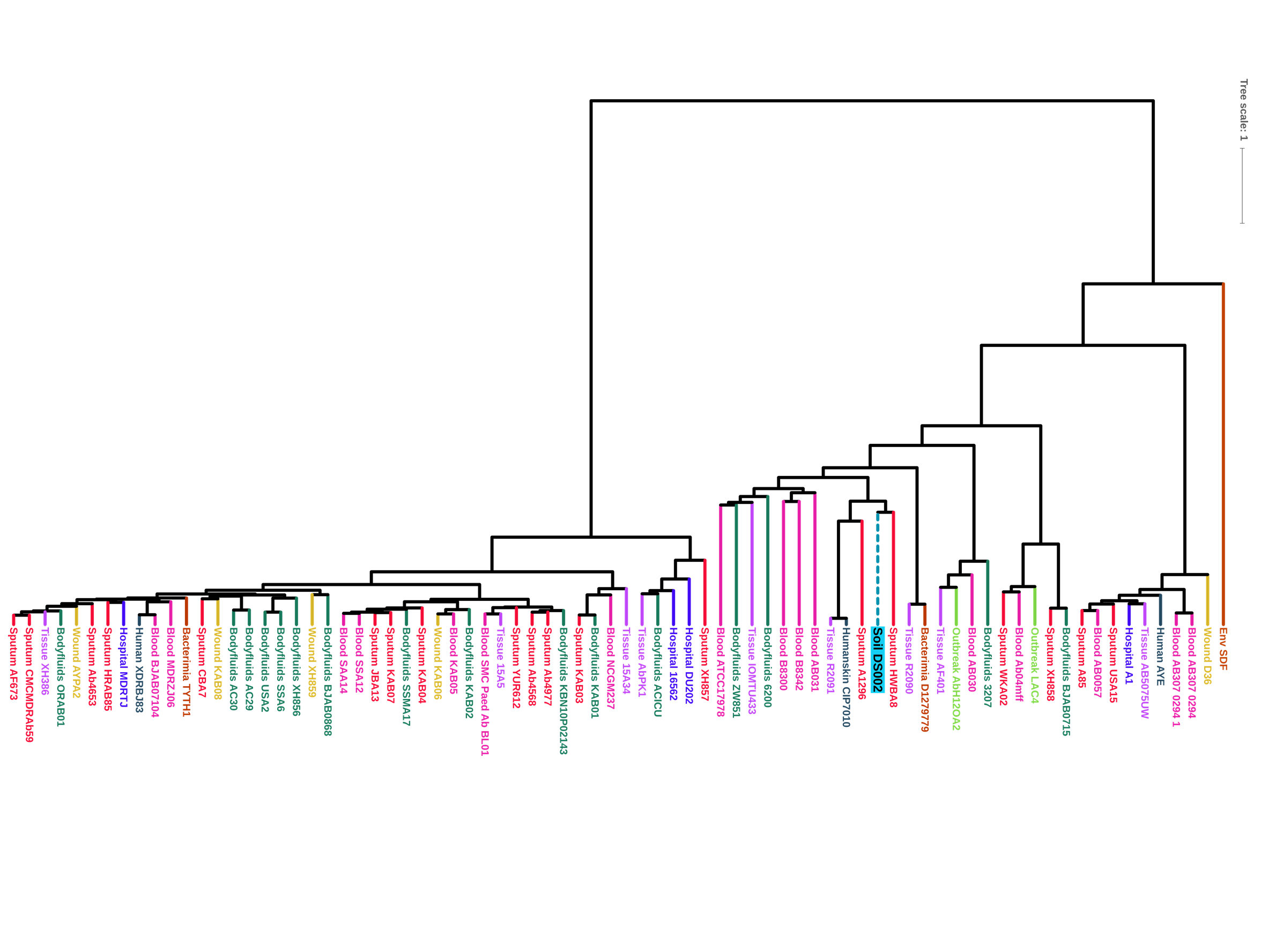

Supplement: S3 Fig — Strains with similar isolation source are shown with identical colors. The dotted blue line indicates the clade of soil isolate DS002 highlighted in blue. (TIF) [file pone.0218204.s004.tif]

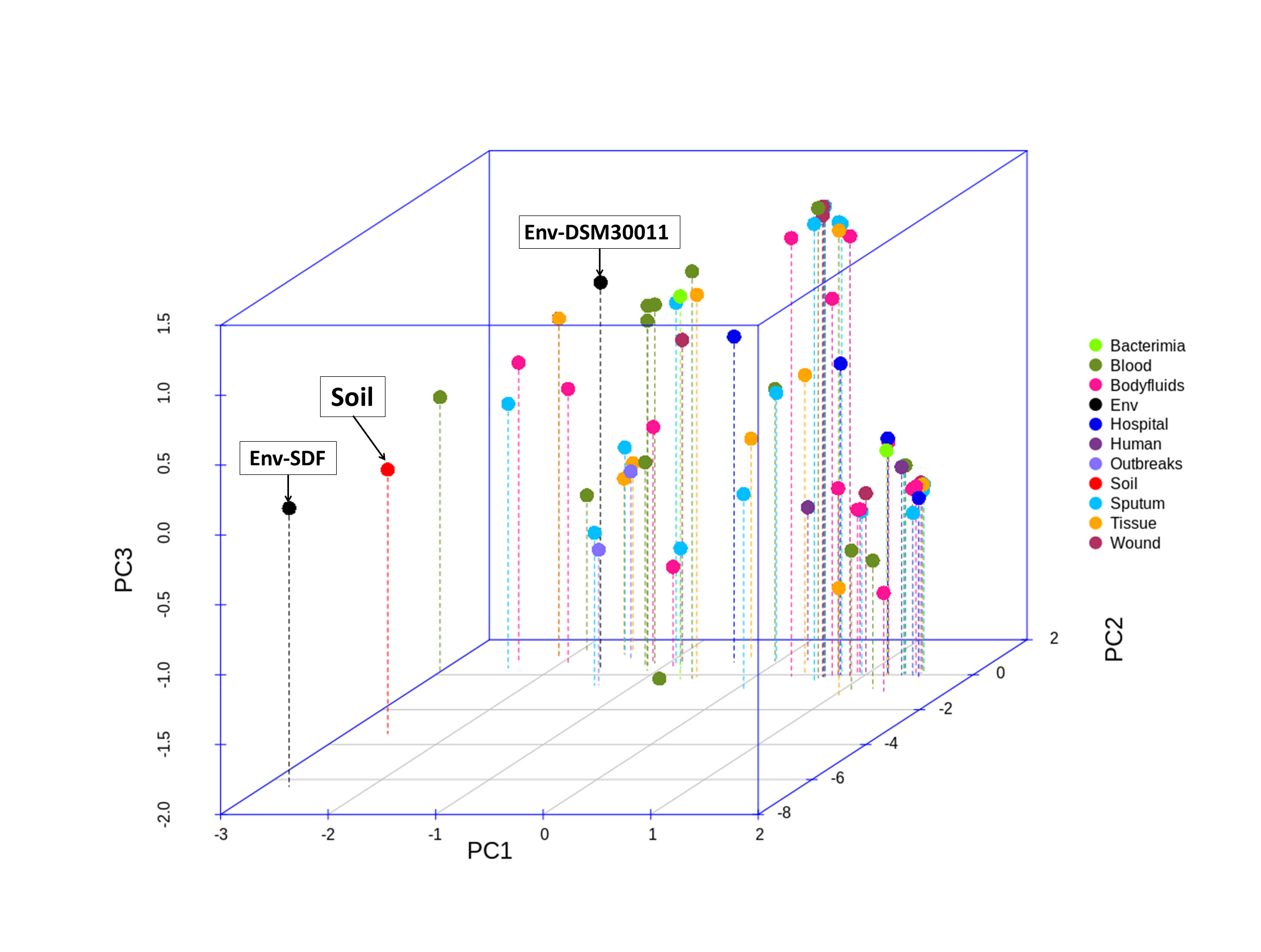

Supplement: S4 Fig — Strains with similar isolation source are shown with identical colors. (TIF) [file pone.0218204.s005.tif]

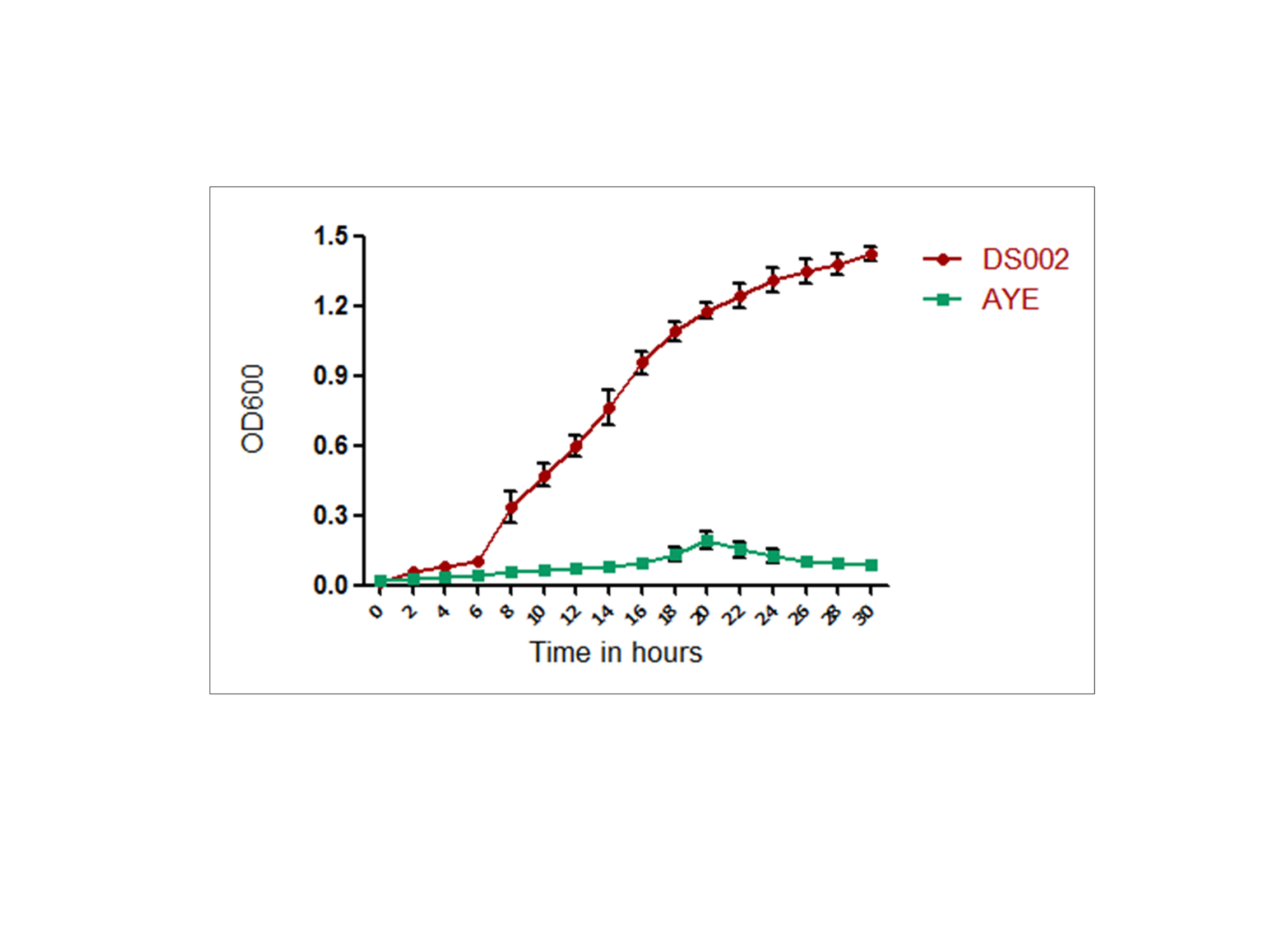

Supplement: S5 Fig — (TIF) [file pone.0218204.s006.tif]

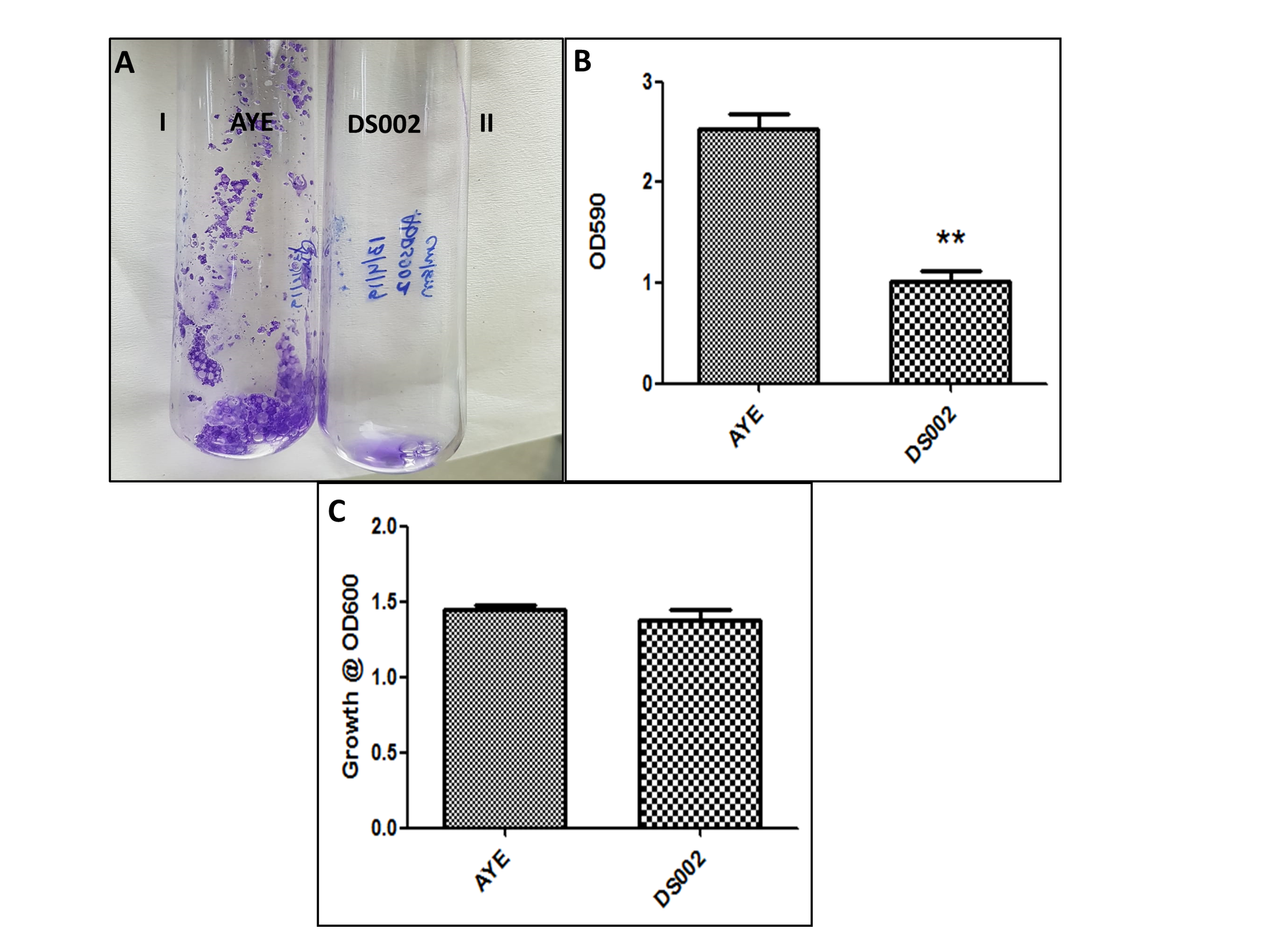

Supplement: S6 Fig — OD590 values obtained for the ethanol extracts prepared from these two tubes indicating the extent of biofilm formation is shown in panel B. Panel C represents the bacterial growth as measured by OD600. (TIF) [file pone.0218204.s007.tif]
