# Supplementary material for: Comparative genome analysis reveals niche-specific genome expansion in Acinetobacter baumannii strains
Source: PLoS One. 2019 Jun 13;14(6):e0218204. doi: 10.1371/journal.pone.0218204 (PMC6563999; doi:10.1371/journal.pone.0218204)
Supplement: S1 Table — (DOCX) [file pone.0218204.s008.docx]

**Table S1: Six circularized contigs obtained by de novo assembly**

| **Contig** | **Assembly length (bp)** | **Circularized length (bp)** | **Reads** | **Coverage** | **CDS** |
| --- | --- | --- | --- | --- | --- |
| 0 | 3434229 | 3430798 | 65341 | 97 | 3569 |
| 2 | 9061 | 4586 | 1053 | 886 | 8 |
| 3 | 147906 | 134338 | 4218 | 171 | 142 |
| 4 | 19631 | 9900 | 416 | 235 | 13 |
| 5 | 21366 | 11291 | 418 | 196 | 16 |
| 12 | 45638 | 37365 | 866 | 129 | 41 |
| Total |  |  | 72343 (90.2%) |  | 3822 |

**Table S1** shows the contigs that are obtained by de novo assembly of the raw reads by Canu+HISEA pipeline. A total of 6 circularized contigs were obtained after the assembly. The largest contig corresponds to the chromosome, whereas the remaining contigs denote the circular plasmids present in the organism.
