# Supplementary material for: Comparative genome analysis reveals niche-specific genome expansion in Acinetobacter baumannii strains
Source: PLoS One. 2019 Jun 13;14(6):e0218204. doi: 10.1371/journal.pone.0218204 (PMC6563999; doi:10.1371/journal.pone.0218204)
Supplement: S2 Table — (DOCX) [file pone.0218204.s009.docx]

**Table S2: The distribution of core, accessory, unique and exclusively absent genes in all the 78 *A. baumannii* strains under study**

| **Genome no.** | **Isolation Source_Strain name** | **No. of core genes** | **No. of accessory genes** | **No. of unique genes** | **No. of exclusively absent genes** |
| --- | --- | --- | --- | --- | --- |
| 1 | Blood_NCGM_237 | 1344 | 1725 | 34 | 115 |
| 2 | Tissue_IOMTU_433 | 1344 | 2119 | 57 | 0 |
| 3 | Body_fluids_ACICU | 1344 | 2113 | 18 | 0 |
| 4 | Blood_AB307_0294_1 | 1344 | 1947 | 5 | 7 |
| 5 | Blood_AB0057 | 1344 | 2175 | 12 | 2 |
| 6 | Hospital_1656_2 | 1344 | 2064 | 7 | 2 |
| 7 | Blood_MDR_ZJ06 | 1344 | 2273 | 23 | 6 |
| 8 | Hospital_MDR_TJ | 1344 | 2148 | 1 | 2 |
| 9 | Blood_BJAB07104 | 1344 | 2185 | 1 | 0 |
| 10 | Body_fluids_BJAB0715 | 1344 | 2252 | 22 | 0 |
| 11 | Body_fluids_BJAB0868 | 1344 | 2166 | 9 | 1 |
| 12 | Bacterimia_TYTH_1 | 1344 | 2084 | 7 | 24 |
| 13 | Bacterimia_D1279779 | 1344 | 1891 | 13 | 2 |
| 14 | Body_fluids_ZW85_1 | 1344 | 1926 | 47 | 0 |
| 15 | Body_fluids_AC29 | 1344 | 2048 | 22 | 3 |
| 16 | Body_fluids_AC30 | 1344 | 2030 | 4 | 1 |
| 17 | Outbreak_LAC_4 | 1344 | 2066 | 35 | 1 |
| 18 | Tissue_AB5075_UW | 1344 | 2125 | 22 | 1 |
| 19 | Blood_AB031 | 1344 | 1872 | 74 | 2 |
| 20 | Blood_AB030 | 1344 | 2226 | 36 | 2 |
| 21 | Outbreak_AbH12O_A2 | 1344 | 2022 | 12 | 5 |
| 22 | Body_fluids_6200 | 1344 | 2009 | 59 | 6 |
| 23 | Tissue_XH386 | 1344 | 2214 | 5 | 0 |
| 24 | Hospital_A1 | 1344 | 2069 | 19 | 0 |
| 25 | Blood_Ab04_mff | 1344 | 2051 | 24 | 3 |
| 26 | Wound_D36 | 1344 | 2205 | 72 | 0 |
| 27 | Body_fluids_KBN10P02143 | 1344 | 2100 | 0 | 3 |
| 28 | Sputum_YU_R612 | 1344 | 2150 | 0 | 1 |
| 29 | Sputum_XH858 | 1344 | 2239 | 13 | 2 |
| 30 | Wound_XH859 | 1344 | 2064 | 5 | 2 |
| 31 | Sputum_XH857 | 1344 | 2016 | 9 | 0 |
| 32 | Body_fluids_XH856 | 1344 | 2046 | 2 | 1 |
| 33 | Body_fluids_3207 | 1344 | 2049 | 65 | 8 |
| 34 | Body_fluids_ORAB01 | 1344 | 1950 | 1 | 22 |
| 35 | Sputum_CMC_MDR_Ab59 | 1344 | 2082 | 0 | 0 |
| 36 | Hospital_DU202 | 1344 | 2265 | 16 | 0 |
| 37 | Body_fluids_KAB01 | 1344 | 2095 | 3 | 1 |
| 38 | Body_fluids_KAB02 | 1344 | 2159 | 0 | 1 |
| 39 | Sputum_KAB03 | 1344 | 2121 | 0 | 0 |
| 40 | Sputum_KAB04 | 1344 | 2121 | 0 | 0 |
| 41 | Blood_KAB05 | 1344 | 2130 | 2 | 0 |
| 42 | Wound_KAB06 | 1344 | 2129 | 0 | 0 |
| 43 | Sputum_KAB07 | 1344 | 2141 | 3 | 0 |
| 44 | Wound_KAB08 | 1344 | 2120 | 5 | 0 |
| 45 | Sputum_HRAB_85 | 1344 | 2168 | 1 | 1 |
| 46 | Tissue_AF_401 | 1344 | 2216 | 36 | 3 |
| 47 | Sputum_AF_673 | 1344 | 2072 | 3 | 2 |
| 48 | Sputum_A1296 | 1344 | 1818 | 60 | 4 |
| 49 | Human_XDR_BJ83 | 1344 | 2106 | 0 | 8 |
| 50 | Blood_ATCC_17978 | 1344 | 2025 | 159 | 0 |
| 51 | Tissue_15A5 | 1344 | 2039 | 1 | 12 |
| 52 | Blood_SAA12 | 1344 | 2101 | 3 | 1 |
| 53 | Blood_SAA14 | 1344 | 2081 | 0 | 0 |
| 54 | Body_fluids_SSMA17 | 1344 | 2060 | 0 | 1 |
| 55 | Sputum_JBA13 | 1344 | 2086 | 0 | 0 |
| 56 | Sputum_CBA7 | 1344 | 2064 | 4 | 10 |
| 57 | Tissue_15A34 | 1344 | 1996 | 11 | 6 |
| 58 | Body_fluids_SSA6 | 1344 | 2016 | 0 | 0 |
| 59 | Body_fluids_USA2 | 1344 | 2032 | 0 | 1 |
| 60 | Sputum_USA15 | 1344 | 2079 | 7 | 4 |
| 61 | Sputum_HWBA8 | 1344 | 2126 | 74 | 2 |
| 62 | Sputum_WKA02 | 1344 | 2016 | 28 | 1 |
| 63 | Blood_B8342 | 1344 | 2033 | 151 | 3 |
| 64 | Blood_B8300 | 1344 | 1941 | 103 | 4 |
| 65 | Sputum_A85 | 1344 | 2251 | 7 | 0 |
| 66 | Wound_AYP_A2 | 1344 | 2146 | 14 | 0 |
| 67 | Tissue_AbPK1 | 1344 | 2143 | 10 | 3 |
| 68 | Sputum_Ab4977 | 1344 | 2135 | 0 | 0 |
| 69 | Sputum_Ab4653 | 1344 | 2035 | 7 | 2 |
| 70 | Sputum_Ab4568 | 1344 | 2175 | 3 | 1 |
| 71 | Blood_AB307_0294 | 1344 | 1959 | 6 | 0 |
| 72 | Blood_SMC_Paed_Ab_BL01 | 1344 | 2111 | 1 | 2 |
| 73 | Soil_DS002 | 1344 | 1034 | 63 | 189 |
| 74 | Human_AYE | 1344 | 2038 | 28 | 4 |
| 75 | Env_SDF | 1344 | 1079 | 128 | 210 |
| 76 | Human_skin_CIP70.10 | 1344 | 2115 | 0 | 0 |
| 77 | Tissue_R2090 | 1344 | 2010 | 22 | 0 |
| 78 | Tissue_R2091 | 1344 | 2118 | 1 | 0 |

**Core and accessory genes-** For a dataset of n genomes, the gene families with members from all of the n genomes are core genes, gene families having members from x genomes, where 1 < x < n, are accessory genes and those having members in any one particular genome of the dataset are unique genes or singletons

**Unique genes-** the orthologous protein families that contain genes exclusively from a specific genome of the dataset

**Exclusively absent genes/proteins**- orthologous families that contain genes from all genomes except one specific genome
